# Synthesis and Reactivity of a Bioinspired Molybdenum(IV) Acetylene Complex

**DOI:** 10.1021/acs.organomet.1c00289

**Published:** 2021-07-05

**Authors:** Madeleine A. Ehweiner, Ferdinand Belaj, Karl Kirchner, Nadia C. Mösch-Zanetti

**Affiliations:** † Institute of Chemistry, Inorganic Chemistry, University of Graz, 8010 Graz, Austria; ‡ Institute of Applied Synthetic Chemistry, Vienna University of Technology, 1060 Vienna, Austria

## Abstract

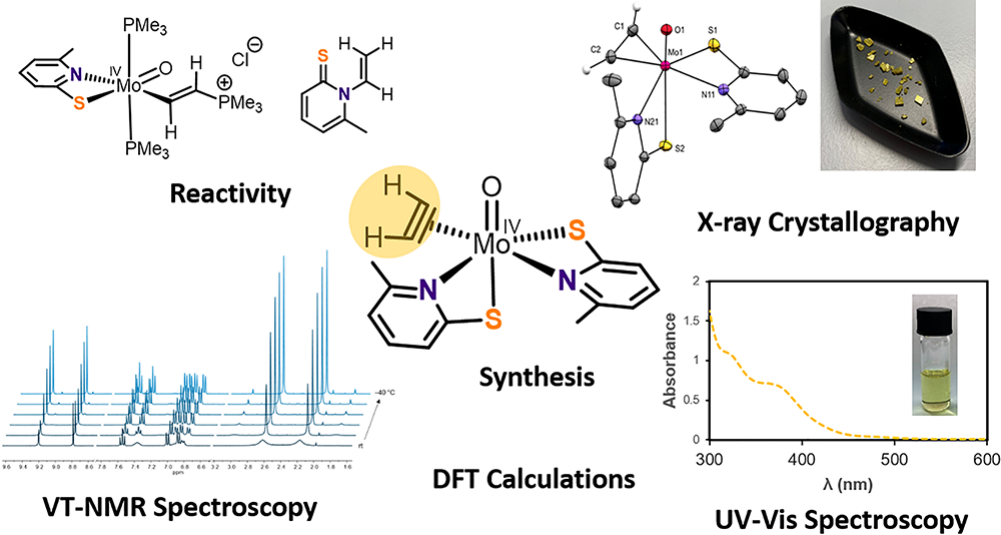

The isolation of a molybdenum(IV) acetylene (C_2_H_2_) complex containing two bioinspired 6-methylpyridine-2-thiolate ligands is reported. The synthesis can be performed either by oxidation of a molybdenum(II) C_2_H_2_ complex or by substitution of a coordinated PMe_3_ by C_2_H_2_ on a molybdenum(IV) center. Both C_2_H_2_ complexes were characterized by spectroscopic means as well as by single-crystal X-ray diffraction. Furthermore, the reactivity of the coordinated C_2_H_2_ was investigated with regard to acetylene hydratase, one of two enzymes that accept C_2_H_2_ as a substrate. While the reaction with water resulted in the vinylation of the pyridine-2-thiolate ligands, an intermolecular nucleophilic attack on the coordinated C_2_H_2_ with the soft nucleophile PMe_3_ was observed to give a cationic ethenyl complex. A comparison with the tungsten analogues revealed less tightly bound C_2_H_2_ in the molybdenum variant, which, however, shows a higher reactivity toward nucleophiles.

## Introduction

Acetylene (C_2_H_2_) is known to be accepted as a substrate by only two enzymes: while the tungstoenzyme acetylene hydratase (AH) catalyzes the hydration of acetylene to acetaldehyde,^1−4^ nitrogenase is capable of reducing acetylene to ethylene (C_2_H_4_).^5−8^ The crystal structure of AH revealed that the octahedral coordination sphere of the tungsten(IV) center in the active site consists of four sulfur atoms from two molybdopterin cofactors, a thiolate from cysteine, and a water molecule.^9^ The mechanism of AH is still under debate, with different suggested mechanisms where H_2_O either stays coordinated to tungsten or is replaced by C_2_H_2_.^9−13^ The molybdenum variant of AH was reported to be 10 times less active in C_2_H_2_ hydration than the tungsten analogue despite exhibiting the same active site architecture as well as a similar protein fold.^14−16^ In contrast to experimental findings, a recent theoretical study suggests the utilization of molybdenum instead of tungsten in bioinspired complexes to be energetically more favorable when a mechanism is considered where C_2_H_2_ is bound to the metal center and subsequently attacked by a hydroxide.^17,18^ As Mo-dependent nitrogenase is known to accept C_2_H_2_ as a substrate, the coordination of C_2_H_2_ to a molybdenum(IV) center was investigated already in the late 1970s.^8,19−22^ However, the coordination of C_2_H_2_ to the bioinspired Mo(IV) complex [MoO(S_2_CNR_2_)_2_] (R = Me, Et) was reported to be reversible, and the formed adduct [MoO(C_2_H_2_)(S_2_CNR_2_)_2_] was found to decompose almost immediately to [Mo_2_O_4_(S_2_CNR_2_)_2_] after exposure to air. Thus, the isolation and characterization by single-crystal X-ray diffraction could not be achieved for the C_2_H_2_ complex, but only for a complex containing a more activated substituted alkyne.^23,24^ Until now, also complexes with C_2_H_2_ coordinated to molybdenum in any other oxidation state remain scarce,^25−28^ with only two mononuclear examples that were characterized by single-crystal X-ray diffraction.^29,30^

Herein, we report the isolation of a Mo(IV) C_2_H_2_ complex containing two bioinspired 6-methylpyridine-2-thiolate (6-MePyS) ligands. We discovered that [MoO(C_2_H_2_)(6-MePyS)_2_] is accessible via two different synthetic pathways, either by oxidation of a Mo(II) C_2_H_2_ complex or by substitution of a coordinated PMe_3_ with C_2_H_2_ on a Mo(IV) center. [MoO(C_2_H_2_)(6-MePyS)_2_] was analyzed by various spectroscopic means and is the first Mo(IV) C_2_H_2_ complex to be characterized by single-crystal X-ray diffraction. A natural population analysis (NPA) and the resulting Wiberg bond indices were used to study the electronic structure and bonding in our C_2_H_2_ complexes to compare them with the previously published tungsten analogues.^31^ Furthermore, the reactivity of the coordinated C_2_H_2_ toward the hard nucleophile H_2_O and the soft nucleophile PMe_3_ was studied.

## Results and Discussion

**Scheme 1:**
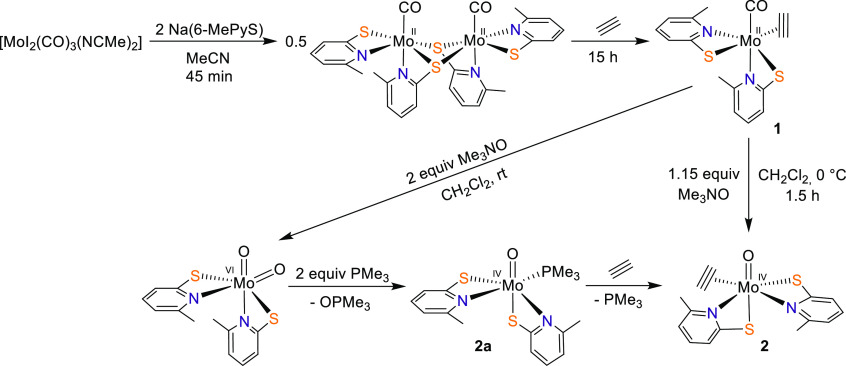
Synthetic Pathways for [MoO(C_2_H_2_)(6-MePyS)_2_] (**2**)

The preparation of the desired Mo(IV) C_2_H_2_ complex follows a two-step procedure. The reaction of [MoI_2_(CO)_3_(NCMe)_2_] with 2.1 equiv of Na(6-MePyS) in MeCN, subsequent addition of acetylene, and workup using silica gel allowed for the isolation of the Mo(II) C_2_H_2_ complex [Mo(CO)(C_2_H_2_)(6-MePyS)_2_] (**1**) in 63% yield (Scheme 1). The intermediate mixture formed after addition of the ligand but prior to purging with acetylene contains different compounds, the main component of which was isolated and identified by a single-crystal X-ray diffraction study as the sulfur-bridged dimer [Mo_2_(CO)_2_(6-MePyS)_4_] (Figures S1 and S2). IR spectroscopy showed two strong carbonyl bands at 1938 and 1852 cm^–1^. This finding is in contrast to the tungsten analogue, where the monomeric [W(CO)_3_(6-MePyS)_2_] was isolated.^31^ Presumably due to the less π basic character of the Mo center, two carbonyl ligands are displaced during the reaction with 6-MePyS. The IR spectrum of **1** shows a strong carbonyl band at 1908 cm^–1^, which is in accordance with the literature values of similar tungsten complexes.^32−34^ Interestingly, the C≡O stretching frequencies of the dithiocarbamate analogue [Mo(CO)(C_2_H_2_)(S_2_CNEt_2_)_2_] and [Mo(CO)(C_2_H_2_)(S_2_P*i*Pr_2_)_2_] are much higher at 1960 and 1950 cm^–1^, respectively, suggesting that our 6-MePyS ancillary ligands provide a more electron rich environment.^25,26^ The sterically hindered C_2_H_2_ protons in **1** resonate at 12.47 and 12.09 ppm in the ^1^H NMR spectrum and thus are shifted upfield in comparison to the tungsten analogue (13.77 and 13.50 ppm). Also, the C_2_H_2_ carbon atoms in the ^13^C NMR spectrum appear to be slightly more shielded in **1** (201.80 and 200.24 ppm for Mo vs 205.73 and 204.14 ppm for W), suggesting that the W-coordinated C_2_H_2_ displays greater double-bond character as a result of tighter bonding.^31^ Similar differences in NMR data are also observed for other Mo(II) and W(II) acetylene complexes, although the C_2_H_2_ protons and carbons appear as singlets in the respective NMR spectra.^26,35^ Recently, we observed the reversible insertion of a second C_2_H_2_ into the W–N bond in [W(CO)(C_2_H_2_)(6-MePyS)_2_] and especially in the unsubstituted analogue [W(CO)(C_2_H_2_)(PyS)_2_].^31,33^ However, when **1** was stirred under a C_2_H_2_ atmosphere for 24 h, insertion of a second C_2_H_2_ did not occur.

**Figure 1:**
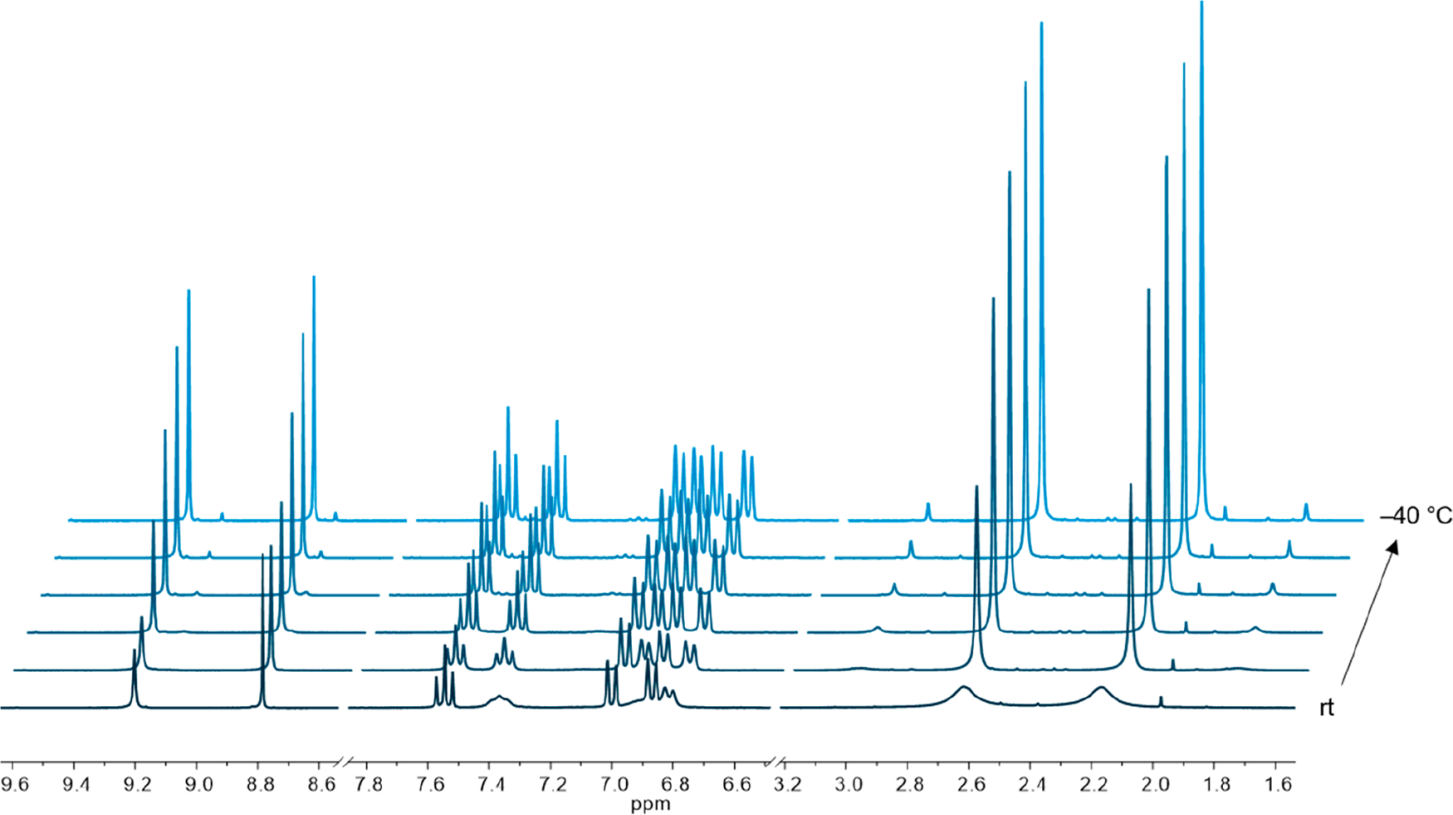
VT ^1^H NMR spectra of **2** in CD_2_Cl_2_ (rt, 0, *–*10, *–*20, *–*30, *–*40 °C).

The desired compound [MoO(C_2_H_2_)(6-MePyS)_2_] (**2**) was synthesized by oxidation of **1** with trimethylamine *N*-oxide in CH_2_Cl_2_ at 0 °C (Scheme 1) and obtained as a yellow crystalline solid in 64% yield after crystallization from MeCN at −25 °C. The Mo(VI) species [MoO_2_(6-MePyS)_2_] and 2,2′-bis(6-methylpyridyl) disulfide were detected as byproducts by NMR spectroscopy, especially when considerably more than 1 equiv of the oxidizing agent was used and the reaction was performed at room temperature. When more than 2 equiv of trimethylamine *N*-oxide was used, **1** was selectively converted to [MoO_2_(6-MePyS)_2_].^36^ Compound **2** exhibits unusually high solubility in CH_2_Cl_2_ and is also well soluble in MeCN, which might be the reason for obtaining **2** only in moderate yield. The IR spectrum shows one strong band at 917 cm^–1^ indicative of ν(Mo=O), which is in accordance with the literature data of similar complexes.^19,20,32,37^ The ^1^H NMR spectrum of **2** in CD_2_Cl_2_ shows a broad singlet for one C_2_H_2_ proton at 9.20 ppm and a sharp singlet for the other at 8.78 ppm, while that of the dithiocarbamate analogue shows only one C_2_H_2_ resonance at 8.73 ppm at room temperature that splits into two singlets at −55 °C.^19,20^ In comparison to the tungsten analogue with resonances at 11.23 and 10.99 ppm, the signals are shifted upfield by 2 ppm, which is much more drastic than that observed in the respective carbonyl complexes (*vide supra*). Furthermore, one set of ligand signals in the aromatic region and resonances of the ligand methyl protons are highly broadened, indicating dynamic behavior at room temperature. To gain more insight into this system, a variable-temperature (VT) ^1^H NMR experiment was performed (Figure 1). When the temperature was lowered to 0 °C, all signals became sharper, indicating a less dynamic behavior, but one of the C_2_H_2_ signals was still broadened. At –10 °C, the resonances were as sharp as those of the tungsten variant at room temperature.^31^ When the temperature was decreased to −20 °C, a second isomer with C_2_H_2_ resonances at 9.13 and 8.77 ppm and CH_3_ resonances at 2.97 and 1.73 ppm was observed. We assume it to be an isomer with respect to the position of the two 6-MePyS ligands. While the dynamic behavior of **2** hampered the recording of a meaningful ^13^C NMR spectrum at room temperature, all resonances were assignable at −10 °C (see the Supporting Information).

**Figure 2:**
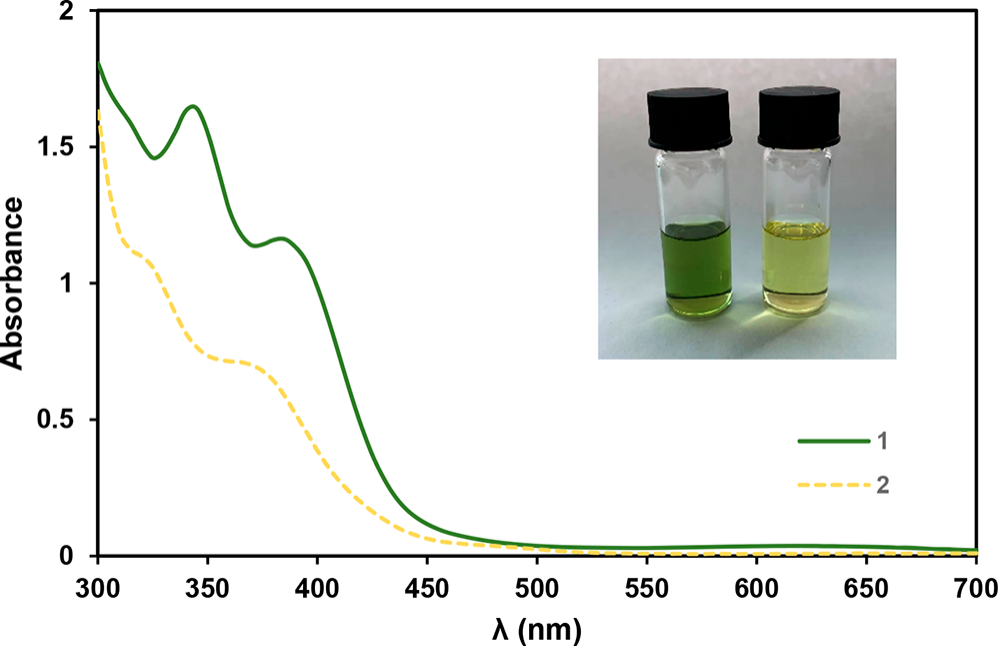
UV*–*vis spectra of **1** and **2** in CH_2_Cl_2_ (*c* = 0.2 mM). Inset: picture of solutions of **1** (left vial) and **2** (right vial) in CH_2_Cl_2_ (*c* = 2.0 mM).

The apparent color change from green to yellow upon oxidation of **1** to **2** is evidenced by the disappearance of the very weak absorption at λ = 620 nm and changes in the high-energy UV–vis region (Figure 2). Both compounds are stable in CH_2_Cl_2_ solutions upon heating to 40 °C for a few hours.

**Figure 3:**
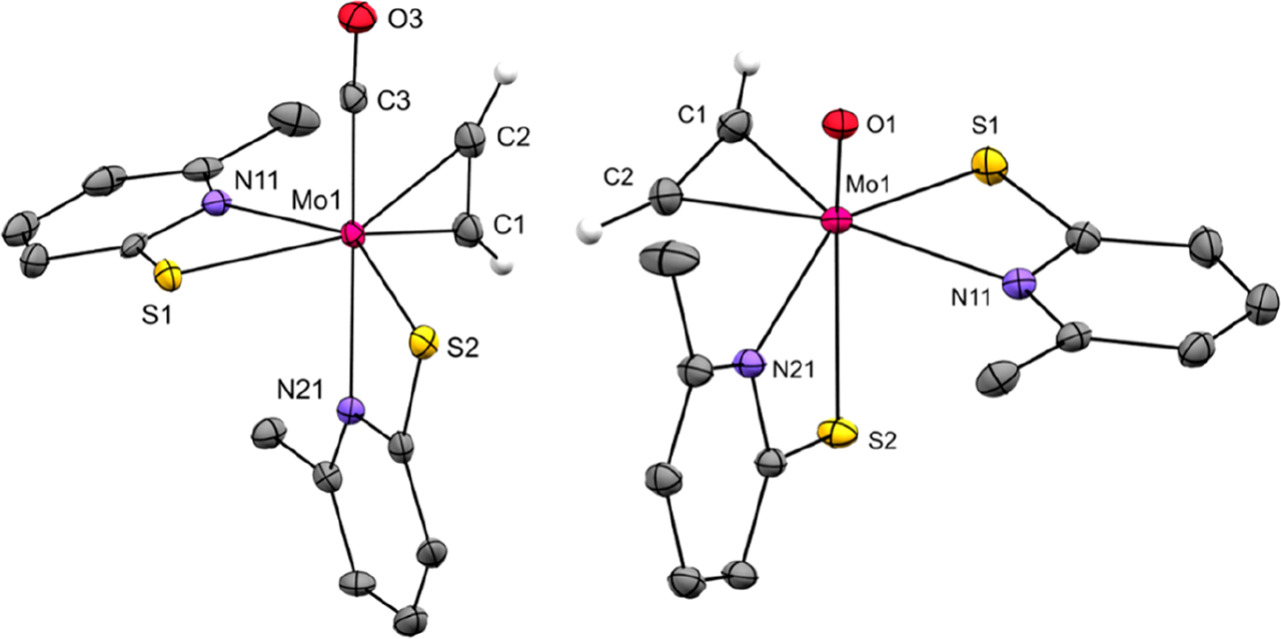
Molecular structures of **1** (left) and **2** (right). Probability ellipsoids are drawn at the 50% probability level. Acetylenic hydrogen atoms are drawn with arbitrary radii, and the others are omitted for clarity.

Single crystals of both **1** and **2** were obtained from CH_2_Cl_2_/heptane solutions at −35 °C, and X-ray diffraction studies unambiguously confirmed their structures (Figure 3). The 6-MePyS ligands exhibit an S,N-trans configuration, and the structures of **1** and **2** and their respective tungsten analogues are isotypic.^31^ The η^2^-C_2_H_2_ group in **2** is opposite to the sulfur of one 6-MePyS and perpendicular to the Mo–O bond with C1–C2–Mo1–O1 90.92(8)°. The C–C bond lengths in **1** and **2** differ only by approximately 0.02 Å (1.289(5) Å for **1** vs 1.2649(17) Å for **2**) and are very similar to those in both [Mo(C_2_H_2_)(CN*t*Bu)_2_(S*t*Bu)_2_] (1.28(2) Å)^29^ and [Mo(C_2_H_2_)(dppe)_2_] (1.265(7) Å).^30^ The Mo–C bonds in **1** are considerably shorter than in **2** (2.015(4) and 2.053(3) Å for **1** vs 2.1044(12) and 2.1076(12) Å for **2**). When **2** is compared with the tungsten variant, the M–C and C–C bond lengths differ only by a maximum of 0.03 Å.^31^ While X-ray data of the analogues are thus quite similar, binding differences are more evident in solution when NMR data are compared (*vide supra*).

The geometries and electronic structures of **1** and **2** and their tungsten analogues were investigated by means of DFT/PBE0 calculations. The optimized structures and corresponding frontier orbitals are presented in Figures S21–S23. A natural population analysis (NPA) and the resulting Wiberg bond indices were used to study the electronic structure and bonding of the optimized species (Table 1). NPA charges indicate that the molybdenum-bound carbon atoms are slightly less nucleophilic than the tungsten analogues, whereas tungsten bears a more positive NPA charge than molybdenum. NPA charges of the C_2_H_2_ carbon atoms are only slightly different when carbonyl and oxido complexes are compared. Interestingly, the charges on the two carbon atoms are almost equally distributed in the oxido complexes, while C_2_H_2_ is rather asymmetrically activated in the carbonyl complexes. Possibly, this explains the shorter reaction time of the nucleophilic attack on tungsten-bound C_2_H_2_ in the carbonyl complex in comparison to the oxido complex.^31^ According to the computed NPA charges, the most electrophilic part of the M–C_2_H_2_ moiety is clearly the metal center. Calculated Wiberg bond indices indicate that approximately two electron pairs are shared by the acetylenic carbon atoms in all four complexes; thus, the initial C≡C bond in the uncoordinated C_2_H_2_ is considerably elongated upon coordination. The strongest M–C bond is present in [W(CO)(C_2_H_2_)(6-MePyS)_2_] and the weakest in **2**. The structure of the M–C_2_H_2_ moiety is presumably more akin to a metallacyclopropene, implying a reduced acetylene (C_2_H_2_^2–^), which is also in accordance with the NPA charges of the carbons. When the (light) yellow color of the oxido complexes is considered, oxidation of the metal center concomitant with reduction of the C_2_H_2_ carbon atoms seems feasible. The C≡C bond is more elongated in the carbonyl complexes than in the oxido analogues, reflecting the more electron rich character of the metal center. Altogether, the theoretical results are well in accordance with experimental data.

**Table 1:** Selected NPA Charges and Wiberg Bond Indices of **1** and **2** and Their Tungsten Analogues

	NPA charge			Wiberg bond index		
complex	M	C1	C2	C1–C2	M–C1	M–C2
[Mo(CO)(C_2_H_2_)(6-MePyS)_2_] (**1**)	0.114	–0.302	–0.273	1.82	0.88	0.83
[W(CO)(C_2_H_2_)(6-MePyS)_2_]	0.328	–0.361	–0.314	1.76	0.92	0.87
[MoO(C_2_H_2_)(6-MePyS)_2_] (**2**)	0.872	–0.285	–0.292	2.07	0.71	0.70
[WO(C_2_H_2_)(6-MePyS)_2_]	1.120	–0.335	–0.346	1.99	0.79	0.76

**Scheme 2:**
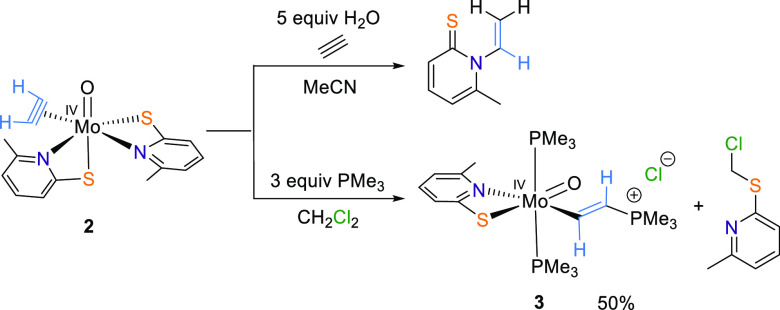
Reaction of **2** with H_2_O (Top) and PMe_3_ (Bottom)

As was previously described, the reaction of [MoO_2_(6-MePyS)_2_] with PMe_3_ gave access to the Mo(IV) complex [MoO(6-MePyS)_2_(PMe_3_)] (**2a**) with one coordinated PMe_3_.^36^ We were interested whether PMe_3_ can be replaced with C_2_H_2_ to synthesize **2**. Therefore, a CH_2_Cl_2_ solution of **2a** was stirred under a C_2_H_2_ atmosphere for up to 20 h, revealing the formation of **2** (Scheme 1) as demonstrated by ^1^H NMR spectroscopy. Although the conversion of **2a** is incomplete and is accompanied by many byproducts, including polyacetylene, the phosphine is in principle replacable by acetylene. However, oxidation of **1** remains the method of choice for the preparation of **2**. With **2** and **2a**, we have two compounds in hand that may qualify as structural models of the molybdenum variant of AH. Especially **2a** could exchange its coordinated PMe_3_ not only with C_2_H_2_ but also with H_2_O, addressing both suggested mechanisms of AH.^38^ When 5 equiv of H_2_O was added to an acetonitrile solution of **2**, no acetaldehyde but an off-white precipitate along with an almost equimolar mixture of **2** and a novel species was detected after 4 days. NMR spectroscopy suggested the latter to be the *N*-vinylated 6-methyl-1-vinylpyridine-2(1*H*)-thione, which is presumably formed after a nucleophilic attack of the pyridine-2-thiolate nitrogen on the C_2_H_2_ followed by protonation of this ethenyl moiety (Scheme 2).^39,40^ This hypothesis is supported by our previous observation that C_2_H_2_ inserts into the W–N bond and not into the W–S bond in [W(CO)(C_2_H_2_)(6-MePyS)_2_], although no insertion has ever been observed in any oxido acetylene complex.^31^ The occurrence of a vinyl moiety is interesting, because in the proposed mechanism of AH, it is also present in ethenol generated from C_2_H_2_ and H_2_O before it tautomerizes to the desired acetaldehyde.^11^ A similar ligand reactivity was reported in the literature when [MoO(HC_2_R)(S_2_CNMe_2_)_2_] was reacted with moisture to yield *trans*-RCH=CH(S_2_CNMe_2_)_2_, which is the analogue to our vinyl compound.^23^ When an acetonitrile solution of **2a** was combined with H_2_O and purged with C_2_H_2_, again the vinyl compound together with an off-white precipitate was formed. The latter was not identified but represents most likely any molybdate species. Addition of triethylamine or pyridine, with the aim of mimicking the acid–base catalysis in the native enzyme, did not substantially alter the reaction outcome but gave the vinylated 6-MePyS more rapidly and more selectively. When D_2_O is added instead of H_2_O, the deuterium is found in the vinyl moiety with equal distribution of the *cis* and *trans* isomers, as observed by ^1^H NMR spectroscopy. Without the addition of water, the vinyl species is not observed at all. We assume that H_2_O does not coordinate to the metal center, since there is no evidence for that by ^1^H NMR spectroscopy, but delivers only the proton for the formation of the vinylated ligand.

Furthermore, we recently reported on the nucleophilic attack on tungsten-bound C_2_H_2_ by the soft nucleophile PMe_3_, forming an ethenyl moiety.^31^ With the molybdenum analogue **2**, we found similar reactivity despite the weaker binding of C_2_H_2_ in the lighter homologue. Thus, addition of 3 equiv of PMe_3_ to a CH_2_Cl_2_ solution of **2** led to an immediate color change from yellow to deep blue-green. NMR spectroscopy undoubtedly revealed the formation of the cationic ethenyl complex [MoO(CHCHPMe_3_)(PMe_3_)_2_(6-MePyS)]Cl (**3**) together with [MoO(6-MePyS)_2_(PMe_3_)] (**2a**) and 6-MePySCH_2_Cl (Scheme 2). After purification by filtration and recrystallization, **3** was isolated as a deep blue-green solid in 50% yield. A nucleophilic attack on coordinated C_2_H_2_ in a molybdenum(IV) complex has not yet been observed. Only an intramolecular nucleophilic attack of a phosphorus atom on coordinated C_2_H_2_ was reported to occur upon oxidation of [Mo(C_2_H_2_)(dppe)_2_] with 2 equiv of [Cp_2_Fe][BF_4_] in THF/MeCN to yield the Mo(II) species [Mo(η^3^-CHCHPPh_2_CH_2_CH_2_PPh_2_-*C*,*C′*,*P*)(dppe)(NCMe)_2_][OTf]_2_.^30^ The formation of **2a**, where C_2_H_2_ is replaced by PMe_3_, confirms the weaker bond of C_2_H_2_ to Mo(IV) in comparison to W(IV), where [WO(PMe_3_)(6-MePyS)_2_] has never been detected. A 2:3 to 1:3 ratio between **2a** and **3** was observed in the crude reaction mixture, with an increasing share of **2a** upon adding less than 3 equiv of PMe_3_. A similar behavior was previously reported for a chromium(0) C_2_H_2_ compound, where addition of a phosphine led either to the substitution of C_2_H_2_ or the formation of an ethenyl complex.^41^ Furthermore, **2** was found to be more reactive toward a nucleophilic attack in comparison to the tungsten analogue, as the reaction was finished after a few minutes, while full conversion of the latter took 7 h under the same conditions.^31^ This difference in reactivity could possibly be explained by more electrophilic C_2_H_2_ carbon atoms and weaker bonds to the metal center in the molybdenum variant.

**Figure 4:**
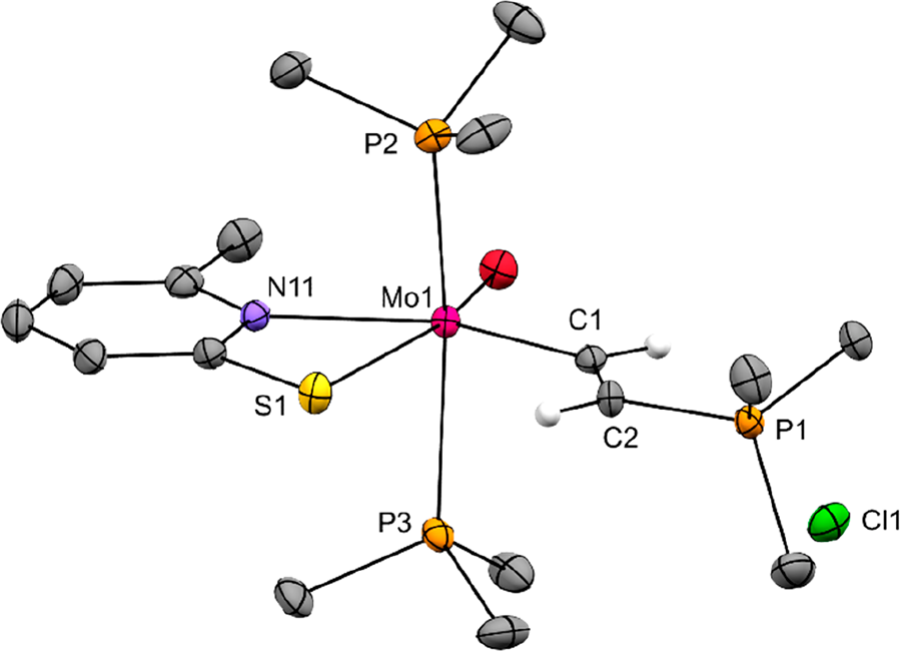
Molecular structure of **3**. Probability ellipsoids are drawn at the 50% probability level. Hydrogen atoms of the ethenyl ligand are drawn with arbitrary radii, and the others are omitted for clarity.

In the ^1^H NMR spectrum of **3** recorded in CD_2_Cl_2_, the ethylene protons give a doublet of doublets at 11.36 and 5.11 ppm with the latter being shifted downfield by approximately 1 ppm in comparison to the tungsten analogue.^31^ In the ^13^C NMR spectrum, the molybdenum-bound ethenyl carbon resonates at 229.0 ppm, while the other gives a doublet at 100.84 ppm. Thus, NMR data are in accordance with previously published data on the tungsten variant.^31^ Single crystals of **3** were easily obtained from a CH_2_Cl_2_/heptane solution at −35 °C, and the structure was unambiguously determined by an X-ray diffraction study (Figure 4). The Mo–C distance of 2.076(2) Å and the C–C distance of 1.355(3) Å in **3** are almost identical with those reported for the tungsten variant.^31^ In comparison to rare examples of molybdenum ethenyl complexes such as [CpMo(CH=CHCO_2_Me)(CO)_2_(PPh_2_Me)] (Mo–C 2.173(6) Å, C–C 1.325(10) Å),^42^ [Me_2_Si(C_5_Me_4_)_2_]Mo(η^2^-*C*,*S*-T) (Mo–C 2.194 Å, C–C 1.197 Å),^43^ and [(κ^2^-CHCHC_6_H_4_S)Mo(PMe_3_)_4_](Mo–C 2.155 Å, C–C 1.240 Å), the Mo–C distance is considerably shorter, while the C–C bond is clearly longer.

## Conclusion

Our studies show that Mo(IV) C_2_H_2_ complexes can be easily isolated and characterized when they are provided with a suitable ancillary ligand system. NMR data and reactivity studies suggest that the coordinated C_2_H_2_ is less tightly bound in the molybdenum variant. The exchange of H_2_O with C_2_H_2_ in the resting state of the active site of AH could be the reason for the Mo variant to be less active if a first-shell mechanism is considered. We showed that Mo-coordinated C_2_H_2_ is more reactive toward nucleophiles due to more electrophilic carbon atoms that are not as tightly bound to the metal center as in the tungsten analogue. We also demonstrated the first nucleophilic attack on a Mo(IV)-bound C_2_H_2_ using PMe_3_ to form a cationic ethenyl complex, while reactions with H_2_O have so far only led to a vinylated ligand. NPA charges indicate that the most electrophilic site of the M–C_2_H_2_ fragment is the metal center in all four investigated C_2_H_2_ complexes, which is presumably the reason why PMe_3_ shows a tendency to bind to the molybdenum center instead of attacking C_2_H_2_ and oxygen-based nucleophiles have not yet made their way to C_2_H_2_ coordinated to group 6 metals. Thus, the key to a nucleophilic attack on Mo- or W-coordinated C_2_H_2_ is to render the carbon atoms more electrophilic by varying either the ancillary ligand system or the oxidation state of the metal center.

## Experimental Section

### Materials and Methods

All synthetic manipulations were performed under a nitrogen atmosphere using standard Schlenk and glovebox techniques. Solvents were purified via a Pure Solv Solvent Purification System. Chemicals were purchased from commercial sources, and apart from acetylene, sodium hydride, and trimethylamine *N*-oxide, all were used without further purification. Acetylene 2.6 was purified by bubbling it through water and concentrated H_2_SO_4_ and subsequently dried by passing it through CaCl_2_ and KOH. Trimethylamine *N*-oxide was purified by sublimation. NaH 60% in mineral oil was washed with pentane, yielding pure NaH. Celite was dried at 100 °C prior to use, and silica gel was washed with Et_3_N and subsequently dried *in vacuo* with heating. NMR spectra were recorded on a Bruker Avance III 300 MHz spectrometer. Chemical shifts δ are given in ppm. ^1^H NMR spectra are referenced to residual protons in the solvent and ^13^C NMR spectra to the deuterated solvent peak. Resonances in ^31^P{^1^H} NMR spectra were referenced to phosphoric acid as an external standard. The multiplicities of peaks are denoted as singlet (s), broad singlet (bs), doublet (d), triplet (t), doublet of doublets (dd), or multiplet (m). NMR solvents were stored over molecular sieves. Solid-state IR spectra were measured on a Bruker ALPHA ATR-FT-IR spectrometer at a resolution of 2 cm^–1^. The relative intensities of signals are declared as strong (s), medium (m), weak (w), and very weak (vw). UV–vis spectra were recorded on a Varian Cary 50 spectrophotometer equipped with a VWR thermostat for controlling temperatures using the Varian Cary WinUV software. Measurements were performed at 25 °C in quartz cuvettes (*d* = 10 mm). Electron ionization mass spectroscopy (EI-MS) measurements have been performed with an Agilent 5973 MSD mass spectrometer with a push rod. Elemental analyses (C, H, N, S) were performed at the Department of Inorganic Chemistry at the University of Technology in Graz and at the microanalytical laboratory of the University of Vienna. Values for elemental analyses are given as percentages.

### Syntheses

The ligand 6-methylpyridine-2-thiol was synthesized from 6-methylpyridin-2-amine according to literature procedures.^44,45^ It was then deprotonated with pure NaH in THF to give Na(6-MePyS) in quantitative yield.^36^ The metal precursor complex [MoI_2_(CO)_3_(NCMe)_2_]^46^ was synthesized according to established procedures. [MoO(6-MePyS)_2_(PMe_3_)]^36^ was obtained by a previously published procedure. In the solid state, complexes **1**–**3** are stable under ambient conditions for a few weeks but ought to be stored and handled under a N_2_ atmosphere over a longer period of time.

#### [Mo(CO)(C_2_H_2_)(6-MePyS)_2_] (**1**)

Na(6-MePyS) (927 mg, 6.30 mmol) was added portionwise to a stirred solution of [MoI_2_(CO)_3_(NCMe)_2_] (1.55 g, 3.00 mmol) in 40 mL of MeCN. After 45 min, the resulting suspension was purged with acetylene for 20 min and was then stirred under an acetylene atmosphere for 15 h at rt. Thereafter, all volatiles were removed *in vacuo*. The resulting green-brown solid was suspended in 50 mL of CH_2_Cl_2_, and this suspension was then filtered through silica gel. The volume of the filtrate was reduced to 8 mL, whereupon 2 mL of heptane was added. The resulting precipitate was isolated by filtration, washed with CH_2_Cl_2_ (2 × 2 mL), and eventually dried *in vacuo* to give [Mo(CO)(C_2_H_2_)(6-MePyS)_2_] (756 mg, 63%) as a green microcrystalline powder. ^1^H NMR (CD_2_Cl_2_, 300 MHz): δ 12.47 (s, 1H, C≡CH), 12.09 (s, 1H, C≡CH), 7.51 (t, 1H, pyH-*p*), 7.08 (t, 1H, pyH-*p*), 6.85 (d, 1H, pyH-*m*), 6.75 (d, 1H, pyH-*m*), 6.63 (d, 1H, pyH-*m*), 6.49 (d, 1H, pyH-*m*), 1.92 (s, 3H, CH_3_), 1.30 (s, 3H, CH_3_) ppm. ^13^C NMR (CD_2_Cl_2_, 75 MHz): δ 238.80 (CO), 201.80 (C≡CH), 200.24 (C≡CH), 175.74 (pyC-*o*), 172.87 (pyC-*o*), 158.54 (pyC-*o*), 156.26 (pyC-*o*), 139.10 (pyC-*p*), 136.12 (pyC-*p*), 123.81 (pyC-*m*), 122.55 (pyC-*m*), 120.11 (pyC-*m*), 118.25 (pyC-*m*), 26.63 (CH_3_), 22.15 (CH_3_) ppm. IR (cm^–1^): 1908 (s, C≡O), 1870 (m, C≡O), 1581 (m), 1552 (m), 1429 (m), 1372 (m), 1167 (m), 997 (w), 875 (w), 823 (w), 774 (m), 734 (w), 713 (m). EI-MS (70 eV) *m*/*z*: M^+^ 400.0, [M – CO]^+^ 372.0, [M – CO – C_2_H_2_]^+^ 345.9. Anal. Calcd for C_15_H_14_N_2_OS_2_Mo: C, 45.23; H, 3.54; N, 7.03; S, 16.10. Found: C, 45.11; H, 3.37; N, 6.98; S, 15.87.

#### [MoO(C_2_H_2_)(6-MePyS)_2_] (**2**)

A solution of trimethylamine *N*-oxide (130 mg, 1.73 mmol) in 5 mL of CH_2_Cl_2_ was added via cannulation to a stirred solution of [Mo(CO)(C_2_H_2_)(6-MePyS)_2_] (598 mg, 1.50 mmol) in 15 mL of CH_2_Cl_2_ at 0 °C. After gas evolution had ceased, all volatiles were removed *in vacuo* with cooling below 0 °C. The resulting ocher solid was dissolved in 20 mL of CH_2_Cl_2_, and this brown solution was quickly filtrated through silica gel. The volume of the filtrate was then reduced to 20 mL before 15 mL of MeCN was added. After evaporation to 5–10 mL, the brown solution was left at −25 °C for 1 week. The solid that formed was isolated by filtration, washed with 3 mL of MeCN, and eventually dried *in vacuo* to give [MoO(C_2_H_2_)(6-MePyS)_2_] (370 mg, 64%) as dark yellow crystals. ^1^H NMR (CD_2_Cl_2_, 300 MHz, −10 °C): δ 9.23 (s, 1H, C≡CH), 8.81 (s, 1H, C≡CH), 7.56 (t, 1H, pyH-*p*), 7.40 (t, 1H, pyH-*p*), 7.00 (d, 1H, pyH-*m*), 6.94 (d, 1H, pyH-*m*), 6.88 (d, 1H, pyH-*m*), 6.79 (d, 1H, pyH-*m*), 2.61 (s, 3H, CH_3_), 2.10 (s, 3H, CH_3_) ppm. ^13^C NMR (CD_2_Cl_2_, 75 MHz, – 10 °C): δ 175.48 (pyC-*o*), 173.79 (pyC-*o*), 158.69 (pyC-*o*), 156.17 (pyC-*o*), 139.72 (C≡CH), 139.37 (pyC-*p*), 139.05 (C≡CH), 137.38 (pyC-*p*), 124.62 (pyC-*m*), 123.48 (pyC-*m*), 120.41 (pyC-*m*), 118.67 (pyC-*m*), 25.10 (CH_3_), 21.18 (CH_3_) ppm. IR (cm^–1^): 3125 (w), 3082 (w), 1662 (m), 1587 (m), 1556 (m), 1451 (m), 1430 (m), 1370 (m), 1173 (m), 1151 (m), 917 (s, Mo = O), 894 (m), 882 (m), 771 (s), 694 (s). EI-MS (70 eV) *m*/*z*: [M – C_2_H_2_]^+^ 362.0. Anal. Calcd for C_14_H_14_N_2_OS_2_Mo: C, 43.52; H, 3.65; N, 7.25; S, 16.60. Found: C, 43.71; H, 3.55; N, 7.34; S, 17.06.

#### [MoO(CHCHPMe_3_)(PMe_3_)_2_(6-MePyS)]Cl (**3**)

A solution of [MoO(C_2_H_2_)(6-MePyS)_2_] (154 mg, 0.40 mmol) and PMe_3_ (140 μL, 1.36 mmol) in 7 mL of CH_2_Cl_2_ was stirred for 1.5 h. After evaporation to dryness, the dark green solid was suspended in 12 mL of CH_2_Cl_2_ and 11 mL of toluene. The supernatant solution was isolated by cannulation. Then, the volume of the filtrate was reduced to 8 mL, and the resulting solid was isolated by filtration, washed with 2 × 5 mL of Et_2_O and 5 mL of pentane, and eventually dried *in vacuo* to yield [MoO(CHCHPMe_3_)(PMe_3_)_2_(6-MePyS)]Cl (105 mg, 50%) as a dark blue-green crystalline solid. ^1^H NMR (CD_2_Cl_2_, 300 MHz): δ 11.36 (dd, *J* = 19.0, 37.8 Hz, 1H, CH), 7.18 (t, *J* = 7.8 Hz, 1H, pyH-*p*), 6.86 (d, *J* = 7.5 Hz, 1H, pyH-*m*), 6.59 (d, *J* = 8.0 Hz, 1H, pyH-*m*), 5.11 (dd, *J* = 18.6, 37.5 Hz, 1H, CH), 2.55 (s, 3H, CH_3_), 2.00 (d, ^2^*J*_PH_ = 13.6 Hz, 9H, PCH_3_), 1.33 (t, ^2^*J*_PH_ = 4.1 Hz, 18H, MoPCH_3_) ppm. ^31^P NMR (CD_2_Cl_2_, 121 MHz): δ 3.06 (bs, PMe_3_), –7.54 (bs, MoPMe_3_) ppm. ^13^C NMR (CD_2_Cl_2_, 75 MHz): δ 229.00 (MoCH), 168.92 (t, *J* = 2.2 Hz, pyC-*o*), 156.30 (pyC-*o*), 135.75 5.11(pyC-*p*), 124.20 (pyC-*m*), 118.06z (pyC-*m*), 100.84 (d, ^1^*J*_CP_ = 75.8 Hz, CH), 24.96 (CH_3_), 14.14 (t, ^1^*J*_CP_ = 13.2 Hz, 6C, MoPCH_3_), 11.86 (d, ^1^*J*_CP_ = 57.6 Hz, 3C, PCH_3_) ppm. IR (cm^–1^): 2967 (w), 2899 (w), 1581 (w), 1550 (w), 1469 (m), 1448 (m), 1430 (m), 1385 (w), 1280 (m), 1172 (m), 1148 (w), 982 (s), 951 (s), 934 (s, Mo = O), 897 (m), 878 (m), 860 (m), 805 (w), 762 (m), 731 (m), 670 (m). Anal. Calcd for C_17_H_35_NOP_3_SClMo: C, 38.83; H, 6.71; N, 2.66; S, 6.10. Found: C, 38.45; H, 6.66; N, 2.62; S, 5.96.

#### 6-Methyl-1-vinylpyridine-2(1*H*)-thione

A solution of [MoO(6-MePyS)_2_(PMe_3_)] (109 mg, 0.25 mmol) in 15 mL of MeCN was purged with C_2_H_2_ for 30 min before Et_3_N (697 μL, 5.00 mmol) and H_2_O (90 μL, 5.00 mmol) were added. The mixture was then stirred for 15 h. After evaporation to dryness, the solid was resuspended in MeCN. The resulting suspension was filtrated through Celite. After evaporation to dryness, an ocher solid was obtained that contained mostly 6-methyl-1-vinylpyridine-2(1*H*)-thione according to NMR spectroscopy. ^1^H NMR (CD_3_CN, 300 MHz): δ 7.38 (d, *J* = 8.4 Hz, 1H, pyH-*m*), 7.17 (dd, *J* = 8.7, 7.1 Hz, 1H, pyH-*p*), 6.79 (dd, *J* = 15.9, 8.3 Hz, 1H, CH), 6.63 (d, *J* = 7.1 Hz, 1H, pyH-*m*), 5.53 (dd, *J* = 8.3, 0.6 Hz, 1H, CH_2_), 5.26 (dd, *J* = 15.9, 0.8 Hz, 1H, CH_2_), 2.36 (s, 3H, CH_3_) ppm. ^13^C NMR (CD_3_CN, 75 MHz): δ 182.37 (pyC-*o*), 150.81 (pyC-*o*), 137.63 (CH), 135.65 (pyC-*p*), 133.46 (pyC-*m*), 116.33 (CH_2_), 115.40 (pyC-*m*), 23.24 (CH_3_). EI-MS (70 eV) *m*/*z*: M^+^ 151.0, [M – H]^+^ 150.0, [M – CH]^+^ 138.0, [M – C_2_H_2_]^+^ 125.0, [C_6_H_7_N]^+^ 93.0.

### X-ray Diffraction Analysis

Single-crystal X-ray diffraction analyses were carried out on a Bruker AXS SMART APEX-II diffractometer equipped with a CCD detector. All measurements were performed using monochromated Mo Kα radiation from an Incoatec microfocus sealed tube at 100 K. Absorption corrections were performed semiempirically from equivalents. Molecular structures were solved by direct methods (SHELXS-97)^47^ and refined by full-matrix least-squares techniques against *F*^2^ (SHELXL-2014/6).^48^ Crystallographic data, figures, and selected geometric parameters are given in the Supporting Information. CCDC 2070035–2070038 contain supplementary crystallographic data for this paper. These data can be obtained free of charge via www.ccdc.cam.ac.uk/data_request/cif.

### DFT Calculations

The computational results presented have been achieved using the Vienna Scientific Cluster (VSC). Calculations were performed using the Gaussian 09 software package and the PBE0 functional without symmetry constraints. All computational details can be found in the Supporting Information.

## Supporting Information Available

Crystallographic data, NMR spectra of all compounds, and computational details (PDF)

## Accession Codes

CCDC 2070035–2070038 contain the supplementary crystallographic data for this paper. These data can be obtained free of charge via www.ccdc.cam.ac.uk/data_request/cif, or by emailing data_request@ccdc.cam.ac.uk, or by contacting The Cambridge Crystallographic Data Centre, 12 Union Road, Cambridge CB2 1EZ, UK; fax: +44 1223 336033.
