# Supplementary material for: Synthesis and Reactivity of a Bioinspired Molybdenum(IV) Acetylene Complex
Source: Organometallics. 2021 Jul 5;40(15):2576–83. doi: 10.1021/acs.organomet.1c00289 (PMC8356224; doi:10.1021/acs.organomet.1c00289)
Supplement: Supplementary file 1 — om1c00289_si_001.pdf [file om1c00289_si_001.pdf]

Supporting Information

Synthesis and Reactivity of a Bioinspired Molybdenum(IV) Acetylene Complex

Madeleine A. Ehweiner,† Ferdinand Belaj,† Karl Kirchner,‡ Nadia C. Mösch-Zanetti†,*

† *Institute of Chemistry, Inorganic Chemistry, University of Graz, 8010 Graz, Austria*

‡ *Institute of Applied Synthetic Chemistry, Vienna University of Technology, 1060 Vienna, Austria*

*Correspondence: nadia.moesch@uni-graz.at

Table of Contents

1 Crystal Structure Determination S1

2 NMR Spectra S9

3 Computational Details S24

4 References S27

1 Crystal Structure Determination

All the single crystal measurements were performed on a Bruker APEX-II CCD diffractometer at 100 K using Mo K_α radiation with a wavelength of 0.71073 Å from an Incoatec microfocus sealed tube equipped with a multilayer monochromator. Absorption corrections were made semi-empirically from equivalents. The structures were solved by direct methods (SHELXS-97)[1] and refined by full-matrix least-squares techniques against F^2 (SHELXL-2014/6)[2]. A weighting scheme of $w = 1/[\sigma^2(F_o^2)+(aP)^2+bP]$ where $P = (F_o^2+2F_c^2)/3$ was used. The non-hydrogen atoms were refined with anisotropic displacement parameters without any constraints except for $[Mo_2(CO)_2(6\text{-}MePyS)_4]$ (see below). The positions of the H atoms of the ethyne ligands and of the ethenyl group were taken from a difference Fourier map, the C–H distances were fixed to 0.95 Å, and these H atoms were refined without any constraints to the bond angles. Common isotropic displacement parameters were refined for the H atoms of the same ethyne ligands, the H atoms of the ethenyl group were refined with individual isotropic displacement parameters. The H atoms of the pyridine rings were put at the external bisectors of the C–C–C angles at C–H distances of 0.95 Å and common individual isotropic displacement parameters were refined for the atoms of the same ring. The H atoms of the methyl groups were refined with common isotropic displacement parameters for the H atoms of the same group and idealized geometries with tetrahedral angles, enabling rotations around the C–C bonds, and C–H distances of 0.98 Å. Crystal data, data collection parameters and structure refinement details are given in Tables S1 and S2. Further refinement information, structure and bonding parameters, SHELXL .res and .hkl files are given in the deposited CIF file which is available free of charge from The Cambridge Crystallographic Data Centre (CCDC 2070035–2070038).

Crystal Structure Determination of $[Mo_2(CO)_2(6\text{-}MePyS)_4]$. The whole molecule was disordered over two orientations around a two-fold rotation axis (see Figure S2) and was refined with site occupation factors of 0.5. The atoms of the pyridine rings were fitted to regular hexagons with distances of 1.39 Å between the ring atoms. The pyridine rings were refined as rigid bodies and the same anisotropic displacement parameters were used for equivalent C atoms of the methyl groups and of the carbonyl ligands. The C–C bonds to the methyl groups were restrained to have the same lengths.

Crystal Structure Determination of 1. Since racemic twinning was detected and a scale factor between the two components refined to a value not significantly different from 0.5 [e.g. 0.51(5)] a twin matrix (-1 0 0 / 0 -1 0 / 0 0 -1) was applied and the structure refined as a 2-component perfect inversion twin.

Crystallographic Data

Table S1. Crystallographic data and structure refinement for $[Mo_2(CO)_2(6\text{-MePyS})_4]$ and **1.**

**Crystal data**	$[Mo_2(CO)_2(6\text{-MePyS})_4]$	$[Mo(CO)(C_2H_2)(6\text{-MePyS})_2]$ **(1)**
CIF data code	ME168	JW12C
Empirical formula	$C_{26}H_{24}Mo_2N_4O_2S_4$	$C_{15}H_{14}MoN_2OS_2$
Formula weight	744.61	398.34
Crystal description	needle, red	block, green
Crystal size	0.22 x 0.06 x 0.03 mm	0.30 x 0.28 x 0.23 mm
Crystal system	monoclinic,	orthorhombic,
Space group	C 2/c	$P\ 2_1\ 2_1\ 2_1$
a	12.546(7) Å	7.1086(4) Å
b	16.524(9) Å	7.9835(4) Å
c	14.570(7) Å	27.0157(13) Å
β	112.201(13)°	
Volume	2797(2) $Å^3$	1533.18(14) $Å^3$
Z	4	4
Calc. density	1.769 Mg/m^3	1.726 Mg/m^3
F(000)	1488	800
Linear absorption coefficient μ	1.228 mm^{-1}	1.126 mm^{-1}
Max. and min. transmission	0.745 and 0.378	1.000 and 0.778
Unit cell determination	2.47° < Θ < 24.93°	2.66° < Θ < 30.69°
Reflections used	513	9417
**Data collection**		
Θ range for data collection	2.14 to 26.00°	1.51 to 29.99°
Reflections collected/ unique	3601 / 2754	11716 / 4427
Significant unique reflections	1695 with I > 2σ(I)	4211 with I > 2σ(I)
R(int), R(sigma)	0.0580, 0.0950	0.0364, 0.0426
Completeness to Θ_{max}	100.0%	99.8%
**Refinement**		
Data/ parameters/ restraints	2754 / 153 / 6	4427 / 203 / 2
Goodness-of-fit on F^2	1.025	1.092
Final R indices [I > 2σ(I)]	R1 = 0.0619, wR2 = 0.1407	R1 = 0.0315, wR2 = 0.0660
R indices (all data)	R1 = 0.1119, wR2 = 0.1692	R1 = 0.0336, wR2 = 0.0668
Weighting scheme param. a, b	0.0787, 3.6652	0.0176, 1.7263
Largest Δ/σ in last cycle	0.000	0.001
Largest diff. peak and hole	1.374 and -0.975 $e/Å^3$	0.891 and -0.836 $e/Å^3$
**CCDC no.**	2070035	2070036

Table S2. Crystallographic data and structure refinement for **2** and **3**.

**Crystal data**	**[MoO(C_2H_2)(6-MePyS)$_2$] (2)**	**[MoO(CHCHPMe$_3$)(PMe$_3$)$_2$(6-MePyS)]Cl (3)**
CIF data code	ME150	ME239
Empirical formula	$C_{14}H_{14}MoN_2OS_2$	$C_{17}H_{35}MoNOP_3S^+Cl^-$
Formula weight	386.33	525.82
Crystal description	plate, yellow	plate, green
Crystal size	0.31 x 0.20 x 0.15 mm	0.27 x 0.24 x 0.06 mm
Crystal system	orthorhombic	orthorhombic,
Space group	P b c a	P b c a
a	14.8606(8) Å	11.7319(4) Å
b	13.3034(7) Å	18.9668(7) Å
c	15.2611(9) Å	22.9609(7) Å
Volume	3017.1(3) Å^3	5109.2(3) Å^3
Z	8	8
Calc. density	1.701 Mg/m^3	1.367 Mg/m^3
F(000)	1552	2176
Linear absorption coefficient μ	1.142 mm^{-1}	0.894 mm^{-1}
Max. and min. transmission	1.000 and 0.684	0.745 and 0.642
Unit cell determination	2.67° < Θ < 40.96°	2.23° < Θ < 28.19°
Reflections used	9847	7745
**Data collection**		
Θ range for data collection	2.45 to 40.00°	2.15 to 29.00°
Reflections collected/ unique	54278 / 9344	70606 / 6786
Significant unique reflections	7502 with I > 2σ(I)	5360 with I > 2σ(I)
R(int), R(sigma)	0.0601, 0.0400	0.0980, 0.0631
Completeness to Θ_{max}	100.0%	100.0%
**Refinement**		
Data/ parameters/ restraints	9344 / 194 / 2	6786 / 255 / 2
Goodness-of-fit on F^2	1.043	1.043
Final R indices [I > 2σ(I)]	R1 = 0.0290, wR2 = 0.0655	R1 = 0.0357, wR2 = 0.0747
R indices (all data)	R1 = 0.0430, wR2 = 0.0721	R1 = 0.0533, wR2 = 0.0836
Weighting scheme param. a, b	0.0218, 1.6667	0.0000, 4.0465
Largest Δ/σ in last cycle	0.003	0.001
Largest diff. peak and hole	0.736 and -1.170 e/Å^3	0.591 and -0.487 e/Å^3
**CCDC no.**	2070037	2070038

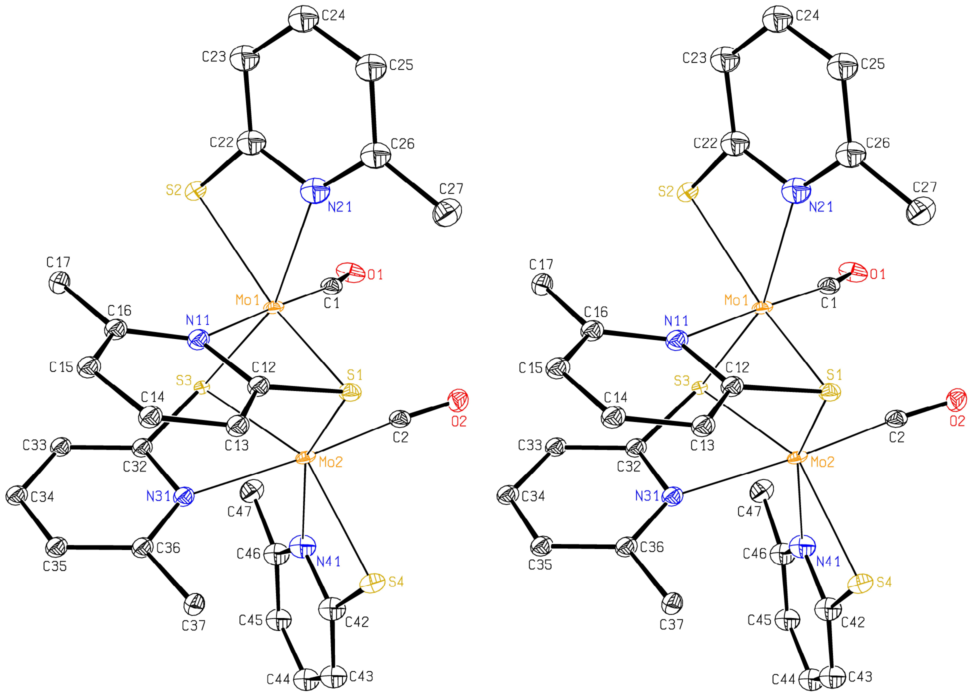

Figure S1. Stereoscopic ORTEP[3] plot of $[Mo_2(CO)_2(6\text{-}MePyS)_4]$ showing the atomic numbering scheme. The probability ellipsoids are drawn at the 30% probability level. The H atoms were omitted for clarity.

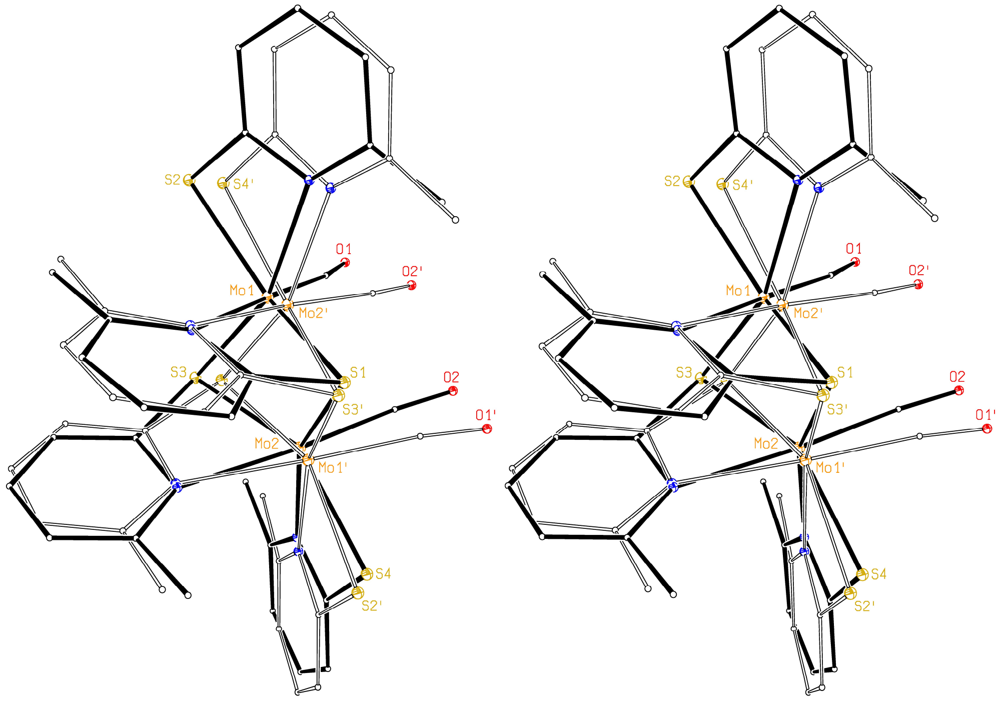

Figure S2. Stereoscopic ORTEP[3] plot of the disorder of $[Mo_2(CO)_2(6\text{-}MePyS)_4]$ around a two-fold rotation axis. The atoms are drawn with arbitrary radii. The H atoms were omitted for clarity.

Table S3. Selected bond lengths (Å) and angles (°) for [$Mo_2(CO)_2(6\text{-}MePyS)_4$].

Mo1–S1	2.437(11)	N41–Mo2–S1	155.7(5)
Mo1–S3	2.403(11)	S3–Mo2–S4	152.4(4)
Mo2–S1	2.418(12)	C12–S1–Mo2	105.2(8)
Mo2–S3	2.419(9)	C12–S1–Mo1	84.7(7)
Mo1–C1	1.990(17)	Mo2–S1–Mo1	66.3(3)
Mo1–N11	2.297(11)	C22–S2–Mo1	80.9(5)
Mo1–N21	2.293(12)	C32–S3–Mo1	106.5(8)
Mo1–S2	2.479(8)	C32–S3–Mo2	83.7(7)
Mo2–C2	1.983(17)	Mo1–S3–Mo2	66.8(2)
Mo2–N31	2.292(11)	C42–S4–Mo2	82.5(5)
Mo2–N41	2.239(12)	C12–N11–Mo1	99.7(7)
Mo2–S4	2.493(8)	C16–N11–Mo1	140.3(7)
C1–O1	1.151(18)	C22–N21–Mo1	96.2(5)
C2–O2	1.150(18)	C26–N21–Mo1	143.7(5)
		C32–N31–Mo2	96.9(7)
C1–Mo1–N11	177.8(7)	C36–N31–Mo2	143.0(7)
N21–Mo1–S3	159.3(4)	C42–N41–Mo2	100.4(6)
S1–Mo1–S2	152.5(4)	C46–N41–Mo2	139.2(6)
C2–Mo2–N31	174.0(6)		

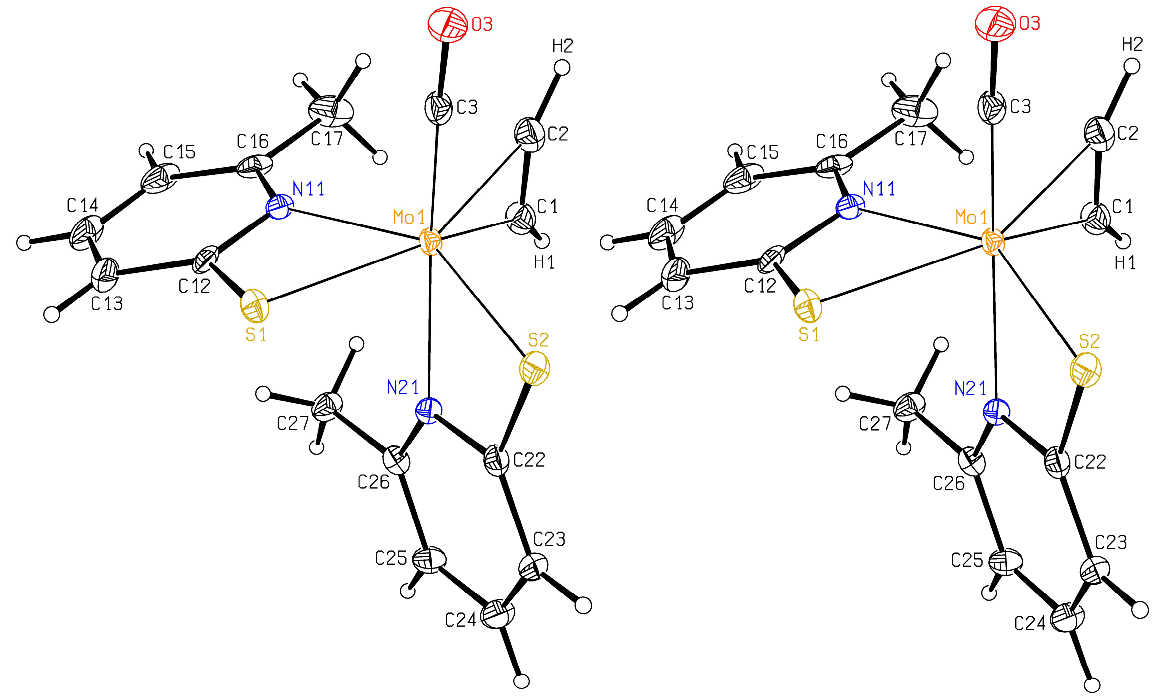

Figure S3. Stereoscopic ORTEP[3] plot of **1** showing the atomic numbering scheme. The probability ellipsoids are drawn at the 50% probability level. The H atoms are drawn with arbitrary radii.

Table S4. Selected bond lengths (Å) and angles (°) for **1**.

Mo1–C1	2.015(4)	C2–Mo1–S1	155.62(11)
Mo1–C2	2.052(3)	N11–Mo1–S2	146.52(10)
Mo1–C3	1.961(4)	C3–Mo1–N21	163.26(14)
Mo1–N11	2.216(3)	O3–C3–Mo1	177.6(4)
Mo1–N21	2.261(3)	C12–S1–Mo1	79.78(13)
Mo1–S1	2.5817(10)	C12–N11–C16	119.8(3)
Mo1–S2	2.3961(10)	C12–N11–Mo1	103.0(2)
C1–C2	1.289(5)	C16–N11–Mo1	136.3(3)
C3–O3	1.160(5)	C22–S2–Mo1	83.63(13)
S1–C12	1.738(4)	C22–N21–C26	120.4(3)
S2–C22	1.764(4)	C22–N21–Mo1	99.2(2)
		C26–N21–Mo1	139.7(3)
S1–Mo1–S2	86.59(3)		
N11–Mo1–N21	92.34(11)	C1–C2–Mo1–C3	-179.1(5)
C1–Mo1–S1	156.51(11)	C2–C1–Mo1–C3	0.9(5)

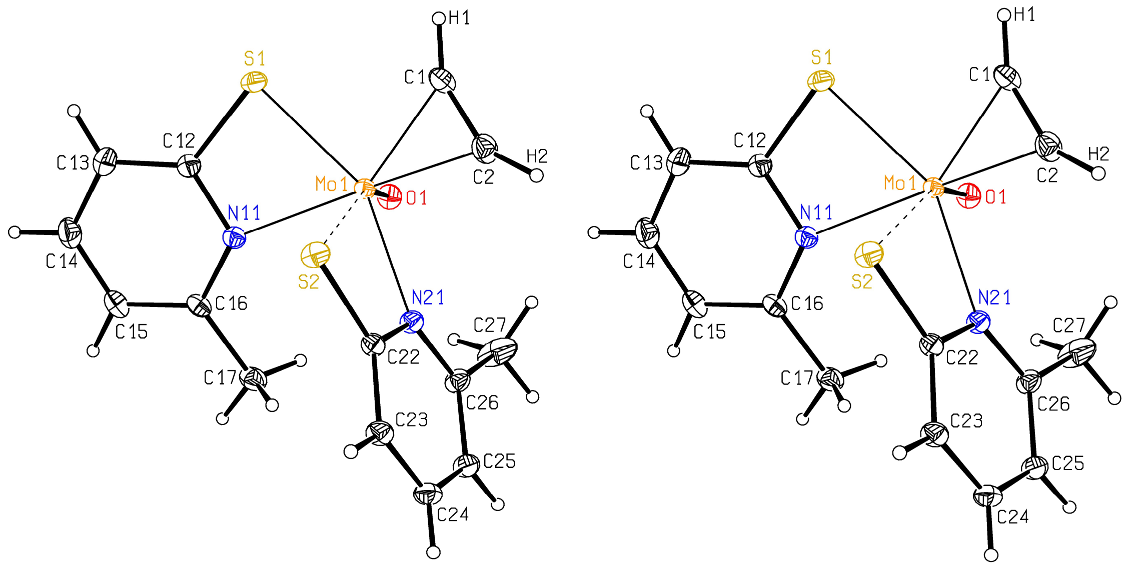

Figure S4. Stereoscopic ORTEP[3] plot of **2** showing the atomic numbering scheme. The probability ellipsoids are drawn at the 50% probability level. The H atoms are drawn with arbitrary radii. The rather long Mo1····S2 bond [2.6264(3) Å *vs.* Mo1–S1 2.4069(3) Å] is drawn with a dashed line.

Table S5. Selected bond lengths (Å) and angles (°) for **2**.

Mo1–O1	1.6972(9)	C1–C2–H2	149.4(10)
Mo1–C1	2.1044(12)	C12–S1–Mo1	84.02(4)
Mo1–C2	2.1076(12)	C16–N11–C12	120.35(11)
Mo1–N11	2.2652(10)	C16–N11–Mo1	139.58(8)
Mo1–N21	2.2236(10)	C12–N11–Mo1	99.66(7)
Mo1–S1	2.4069(3)	C22–S2–Mo1	79.61(4)
Mo1–S2	2.6264(3)	C22–N21–C26	120.26(10)
C1–C2	1.2649(17)	C22–N21–Mo1	104.08(7)
S1–C12	1.7521(12)	C26–N21–Mo1	135.34(8)
S2–C22	1.7294(11)		
		C1–C2–Mo1–O1	90.92(8)
C1–Mo1–N11	146.58(4)	C2–C1–Mo1–O1	-97.12(9)
C2–Mo1–N11	167.25(4)	C1–C2–Mo1–N21	-176.35(9)
N21–Mo1–S1	147.52(3)	C2–C1–Mo1–N21	4.12(9)
O1–Mo1–S2	153.81(3)	C1–C2–Mo1–S1	-25.77(9)
C2–C1–H1	150.4(5)	C2–C1–Mo1–S1	155.91(8)

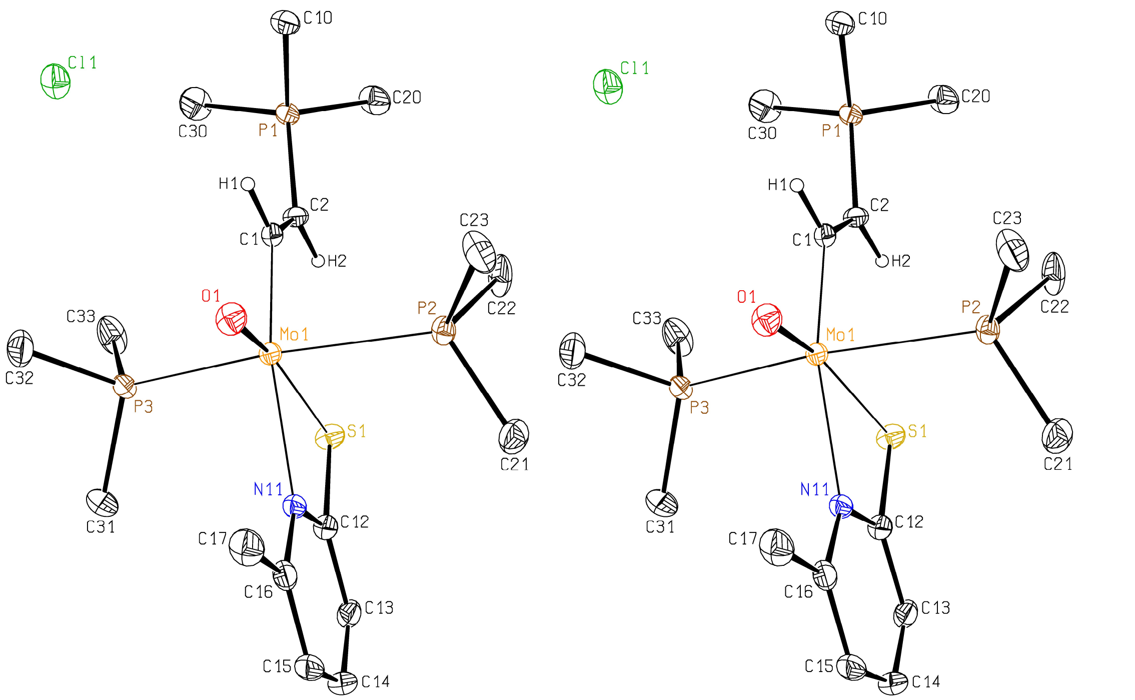

Figure S5. Stereoscopic ORTEP[3] plot of **3** showing the atomic numbering scheme. The probability ellipsoids are drawn at the 50% probability level. The H atoms of the ethenyl group are drawn with arbitrary radii, the other H atoms were omitted for clarity.

Table S6. Selected bond lengths (Å) and angles (°) for **3**.

Mo1–O1	1.6900(16)	C1–Mo1–N11	149.68(8)
Mo1–C1	2.076(2)	P2–Mo1–P3	158.90(2)
Mo1–N11	2.2127(19)	C2–C1–Mo1	135.79(16)
Mo1–P2	2.5012(7)	C2–C1–H1	116.6(6)
Mo1–P3	2.5024(7)	Mo1–C1–H1	107.6(6)
Mo1–S1	2.7136(6)	C1–C2–P1	123.38(16)
C1–C2	1.355(3)	C1–C2–H2	121.1(5)
C2–P1	1.752(2)	P1–C2–H2	115.5(5)
P1–C10	1.775(2)	C12–S1–Mo1	78.46(8)
P1–C30	1.788(3)	C16–N11–C12	120.3(2)
P1–C20	1.793(2)	C16–N11–Mo1	133.04(16)
P2–C21	1.815(3)	C12–N11–Mo1	106.63(15)
P2–C23	1.817(3)		
P2–C22	1.817(2)	Mo1–C1–C2–P1	179.64(13)
P3–C33	1.811(3)	C1–C2–P1–C10	21.5(2)
P3–C32	1.812(3)	C2–C1–Mo1–S1	-1.8(2)
P3–C31	1.822(3)	C2–C1–Mo1–O1	177.2(2)
S1–C12	1.732(2)	C1–Mo1–S1–C12	179.20(11)
		O1–Mo1–N11–C12	-177.82(15)
O1–Mo1–S1	167.45(6)	O1–Mo1–N11–C16	0.7(3)

2 NMR Spectra

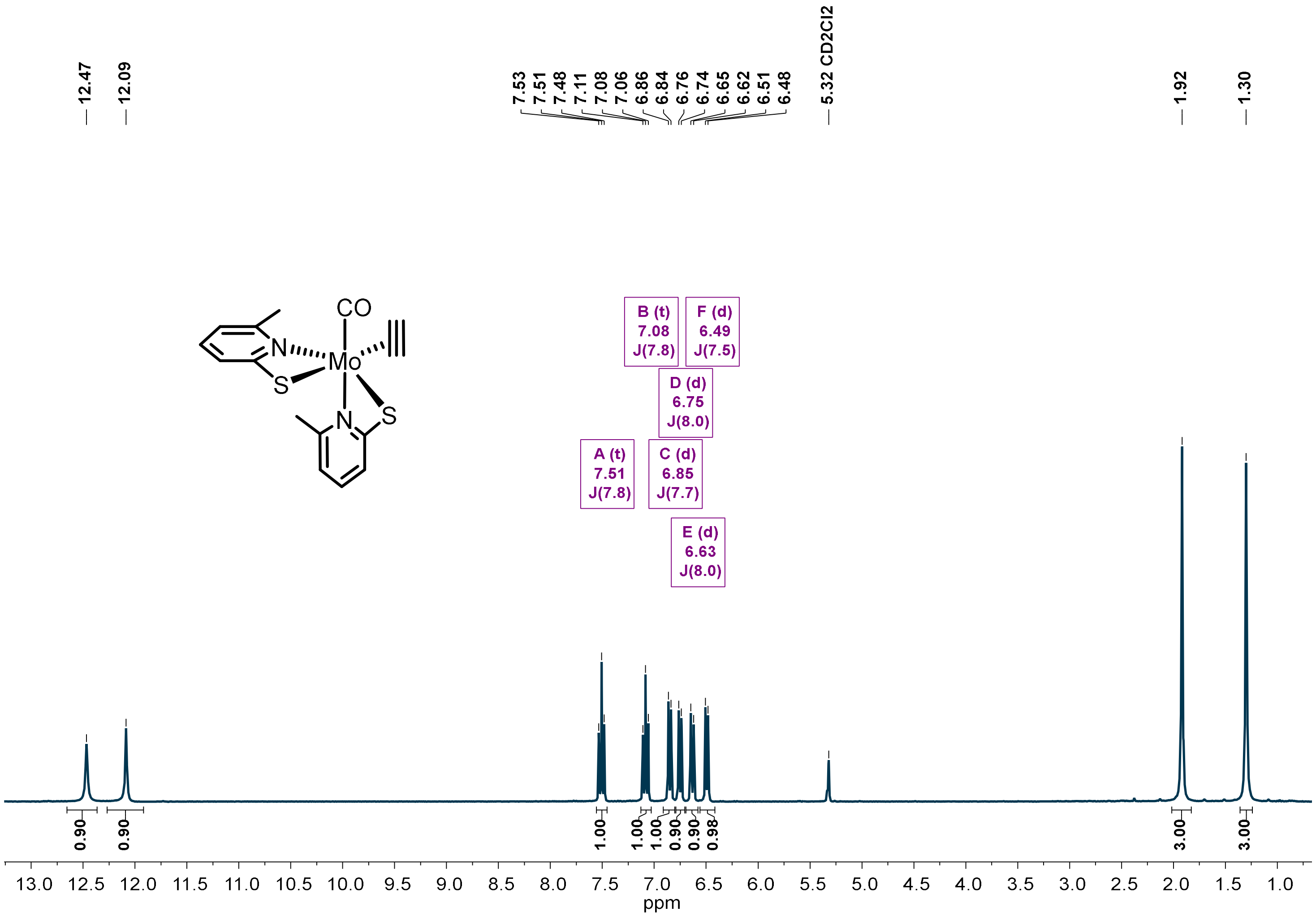

Figure S6. ^{1}H NMR spectrum of **1** in CD_2Cl_2.

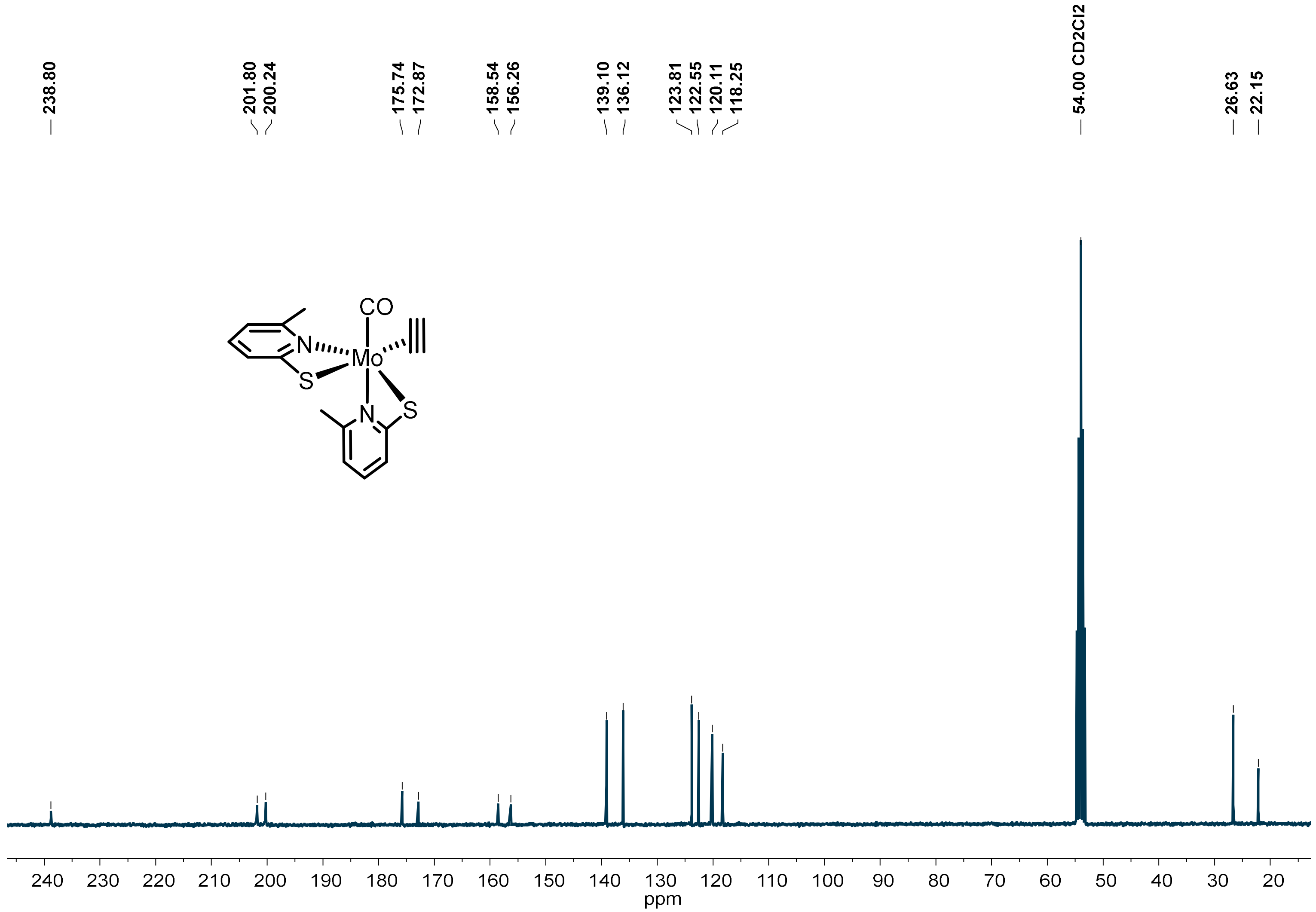

Figure S7. ^{13}C NMR spectrum of **1** in CD_2Cl_2.

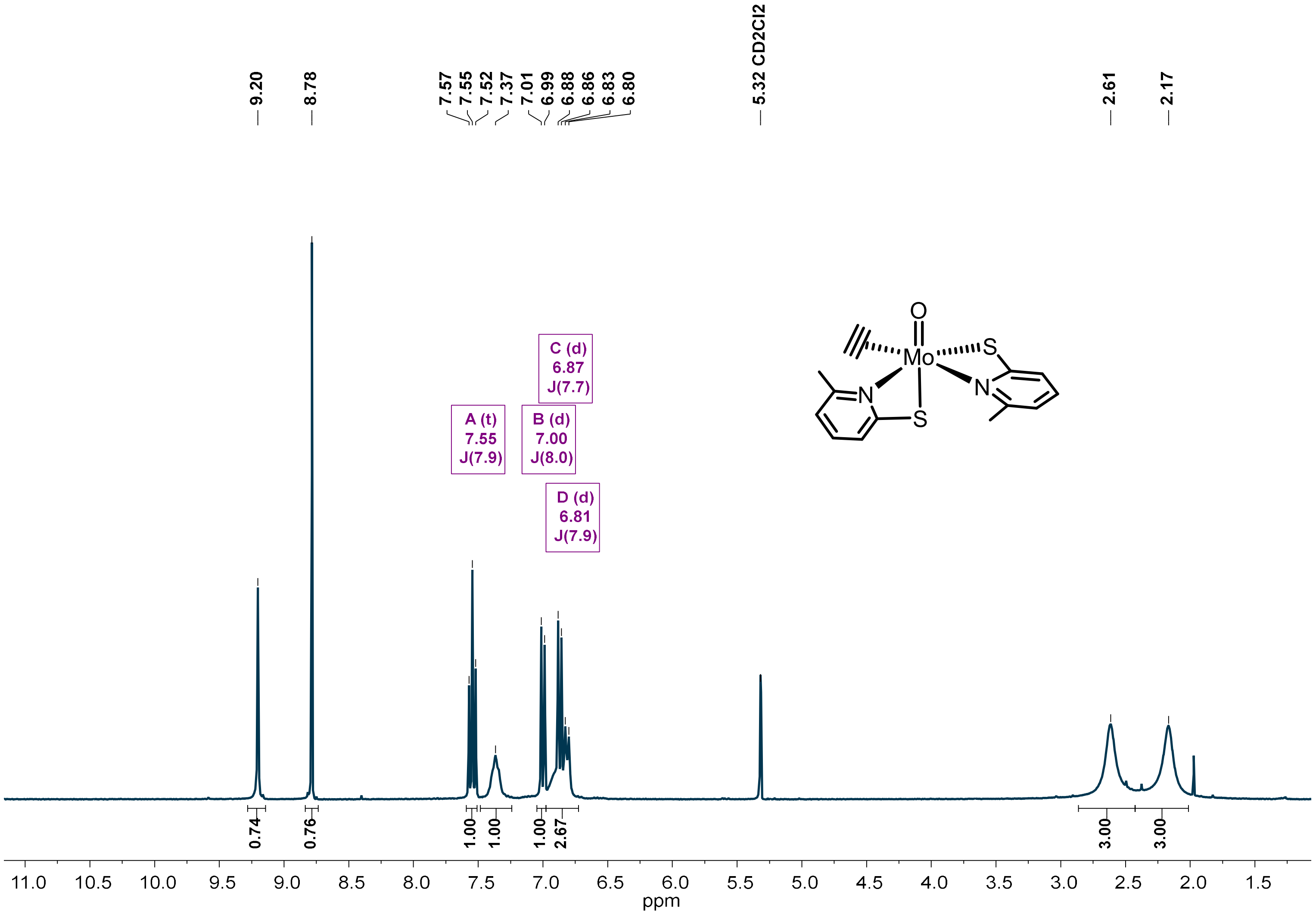

Figure S8. ^{1}H NMR spectrum of **2** in CD_2Cl_2 at 23 °C.

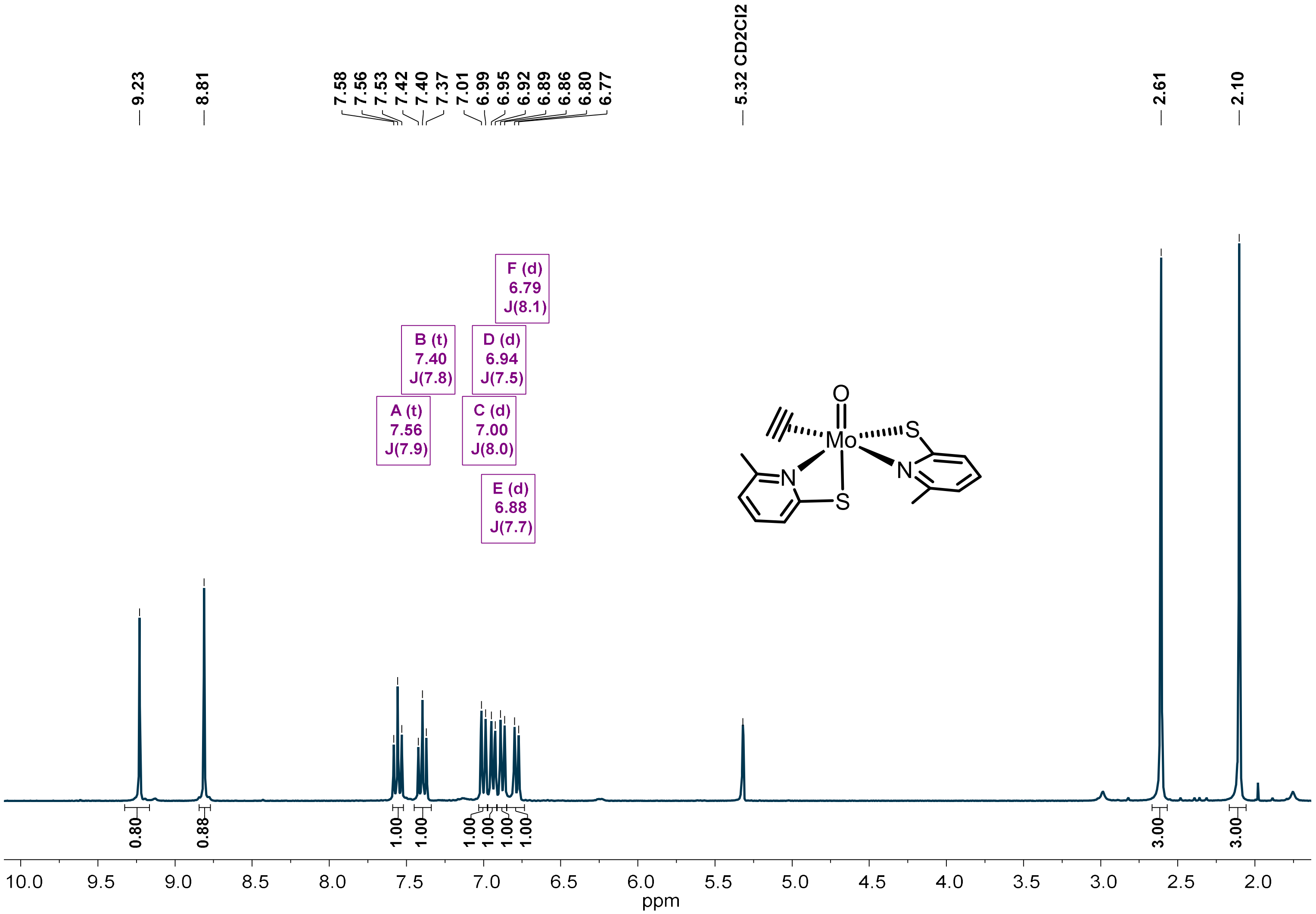

Figure S9. ^{1}H NMR spectrum of **2** in CD_2Cl_2 at –10 °C.

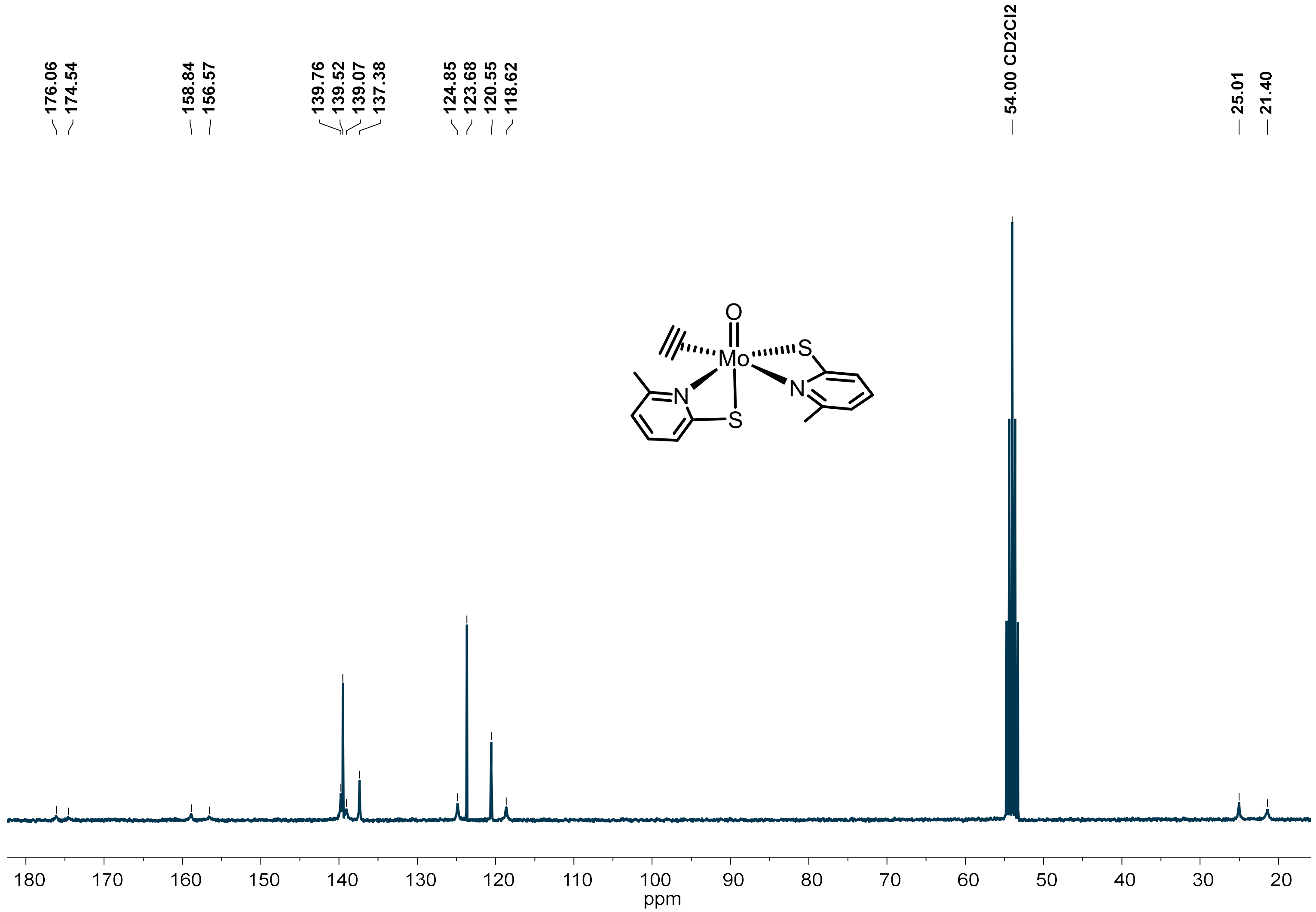

Figure S10. ^{13}C NMR spectrum of **2** in CD_2Cl_2 at 23 °C.

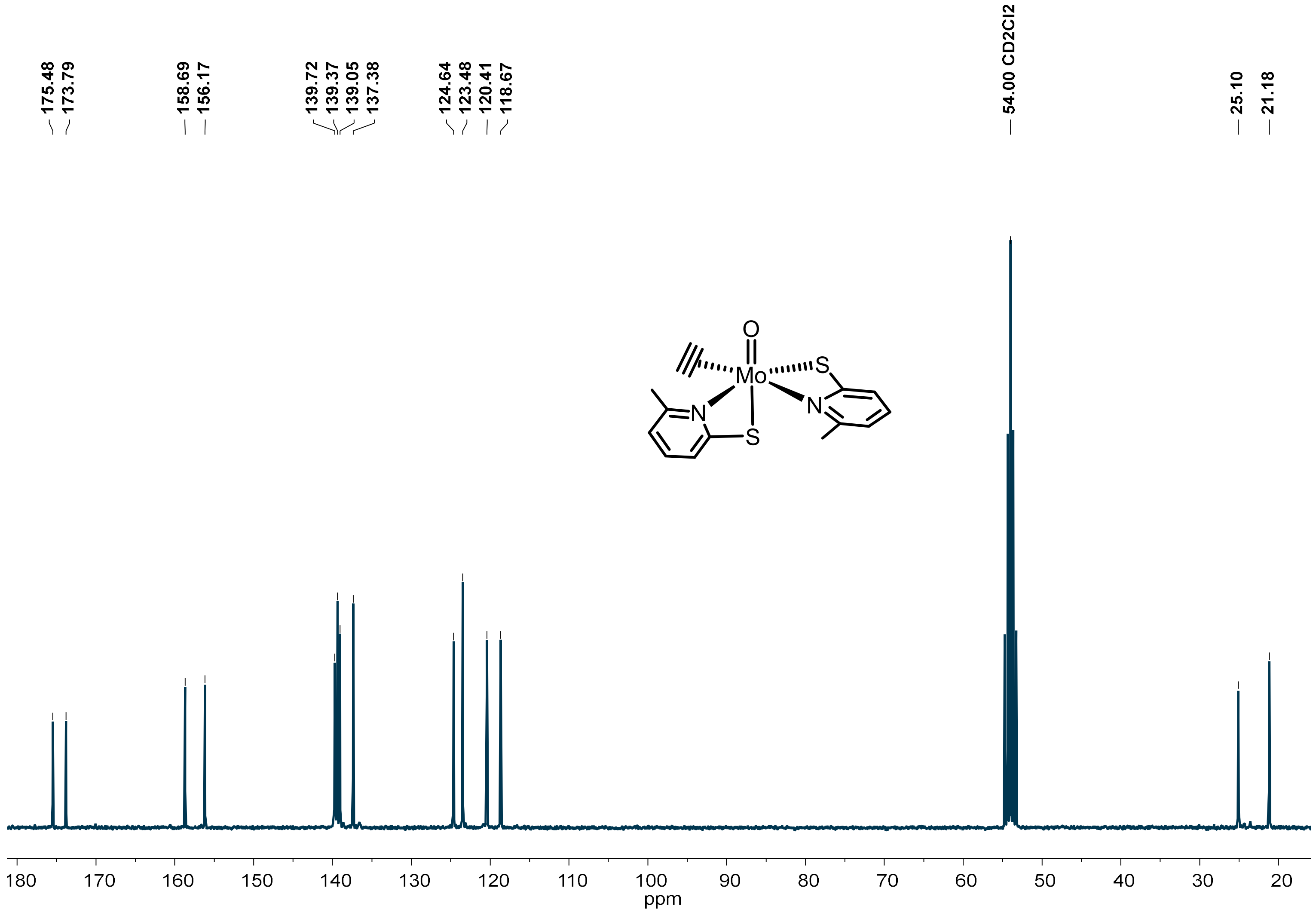

Figure S11. ^{13}C NMR spectrum of **2** in CD_2Cl_2 at –10 °C.

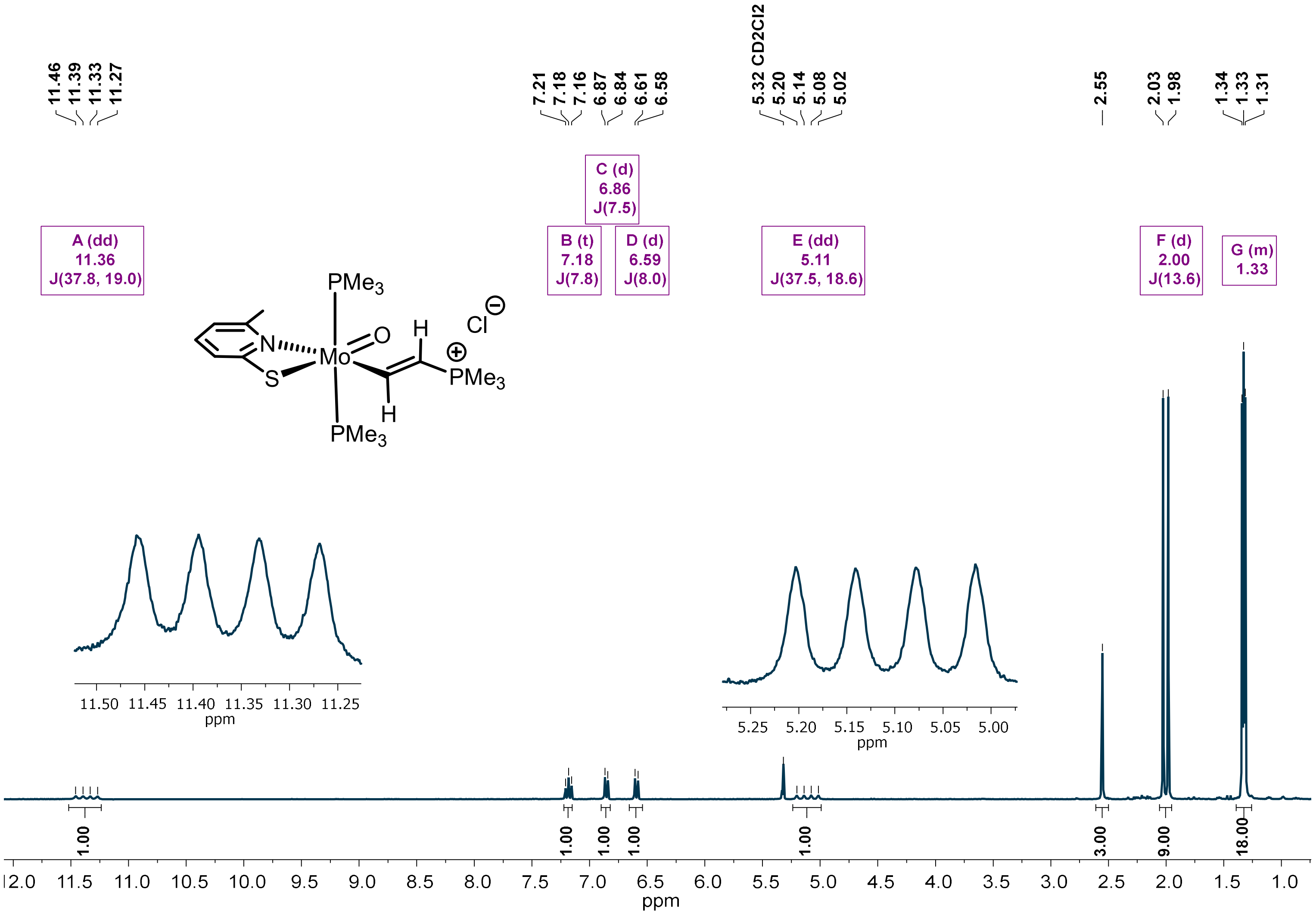

Figure S12. ^{1}H NMR spectrum of **3** in CD_2Cl_2.

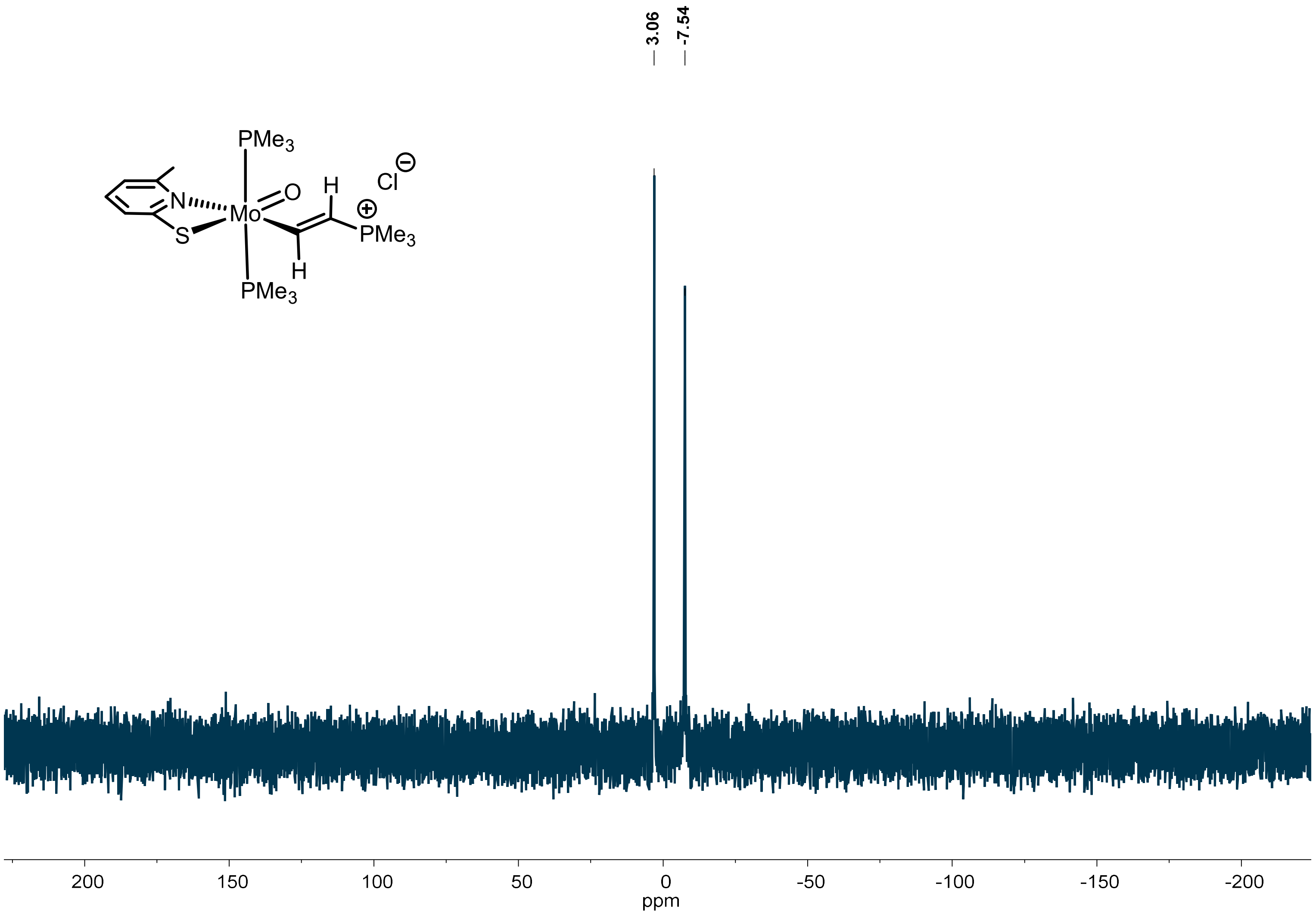

Figure S13. $^{31}P\{^1H\}$ NMR spectrum of **3** in CD_2Cl_2.

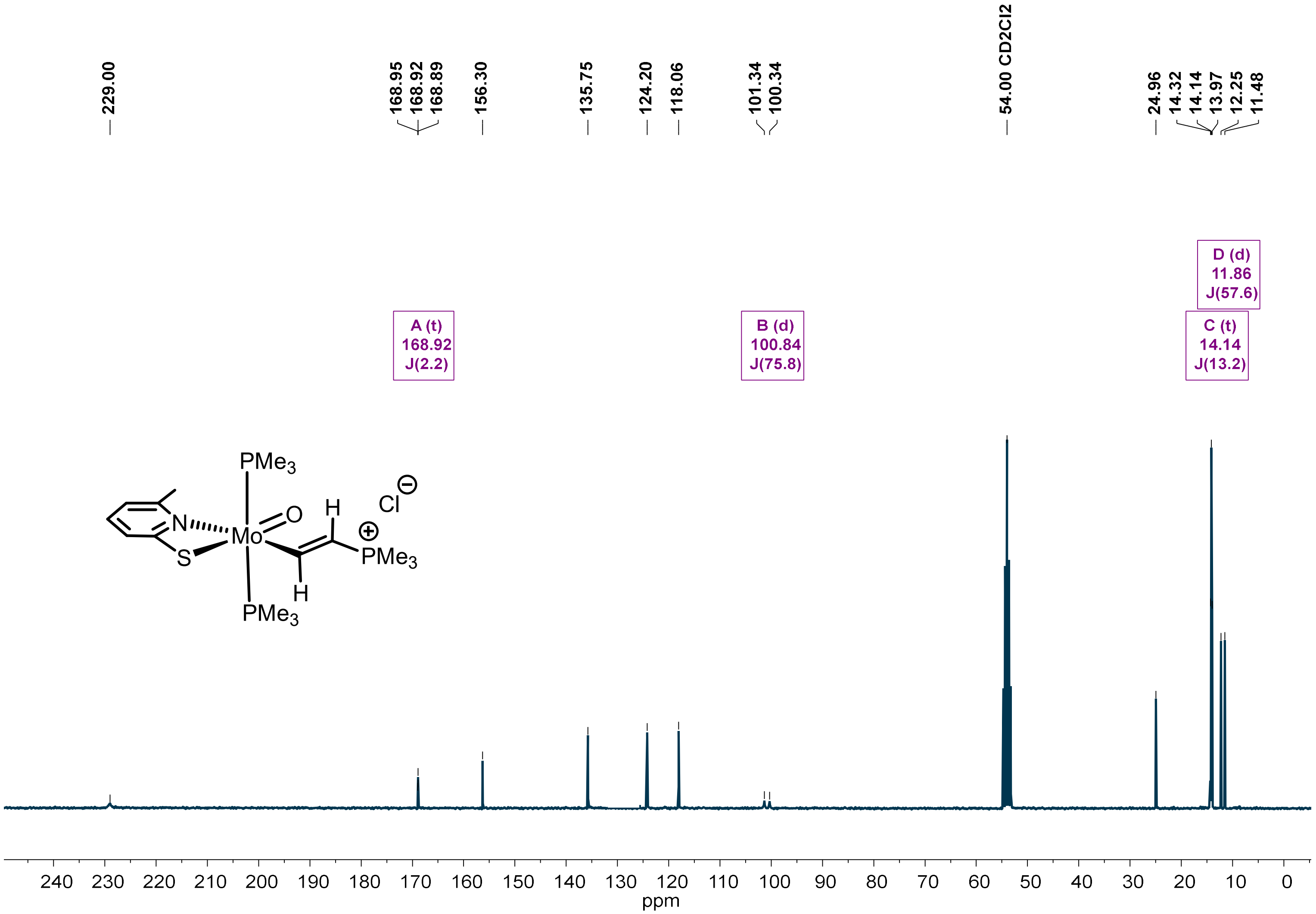

Figure S14. ^{13}C NMR spectrum of **3** in CD_2Cl_2.

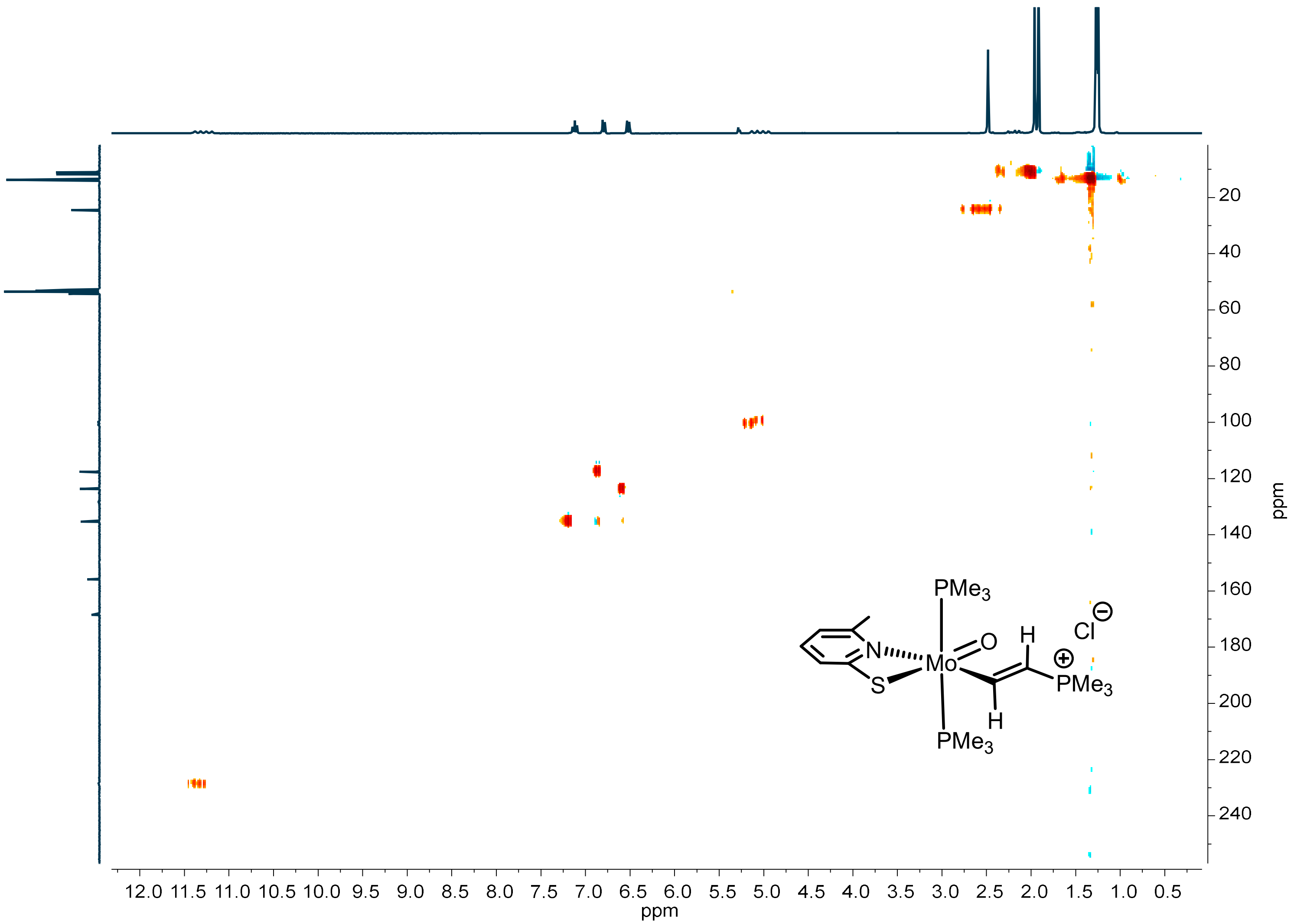

Figure S15. HSQC spectrum of **3** in CD_2Cl_2.

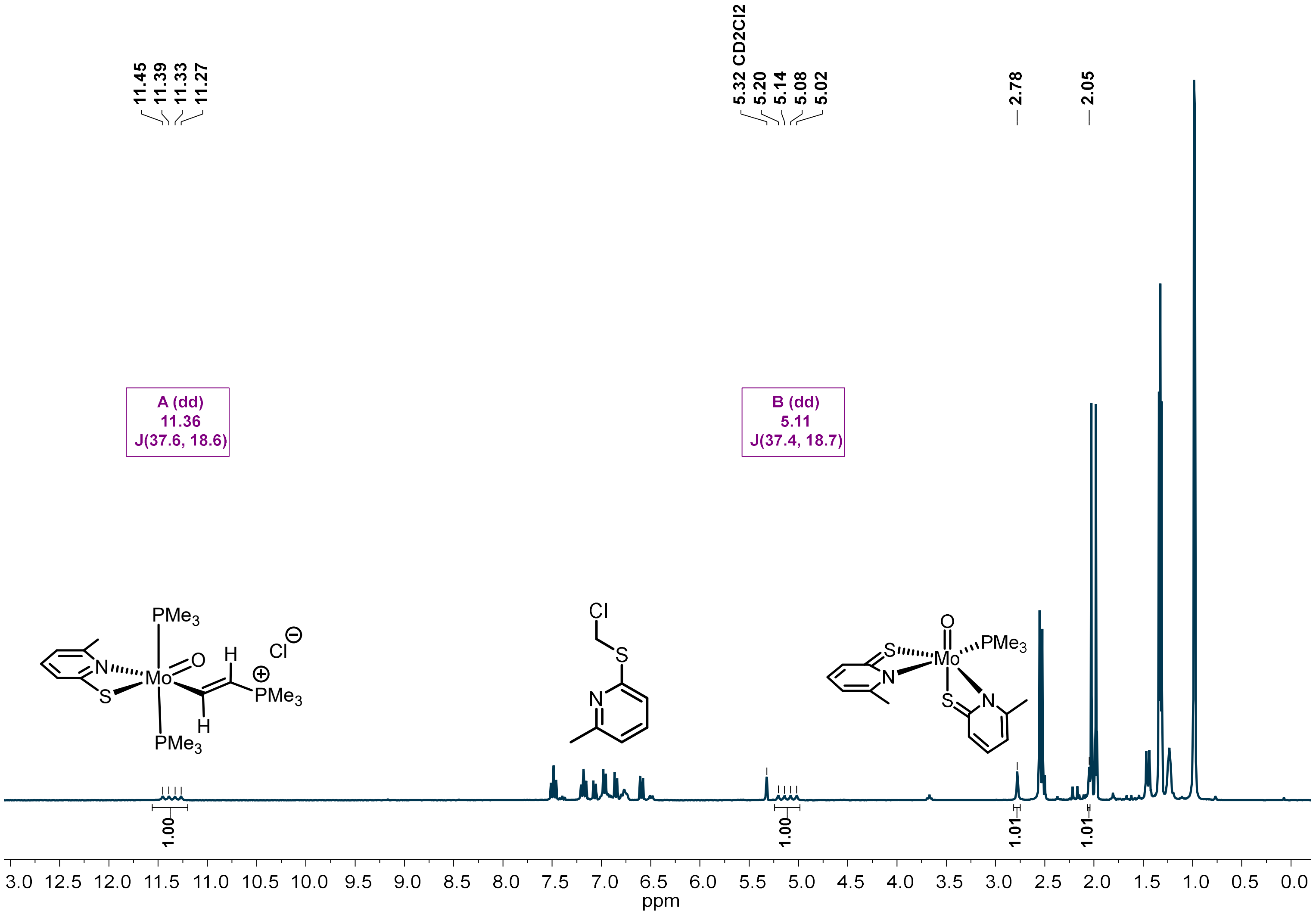

Figure S16. ^{1}H NMR spectrum of a reaction of **2** with 3.5 equiv of PMe_3 in CD_2Cl_2 performed in a J. Young NMR tube. Mixture of mainly **3**, ~25% **2a**, and 6-MePySCH$_2$Cl.

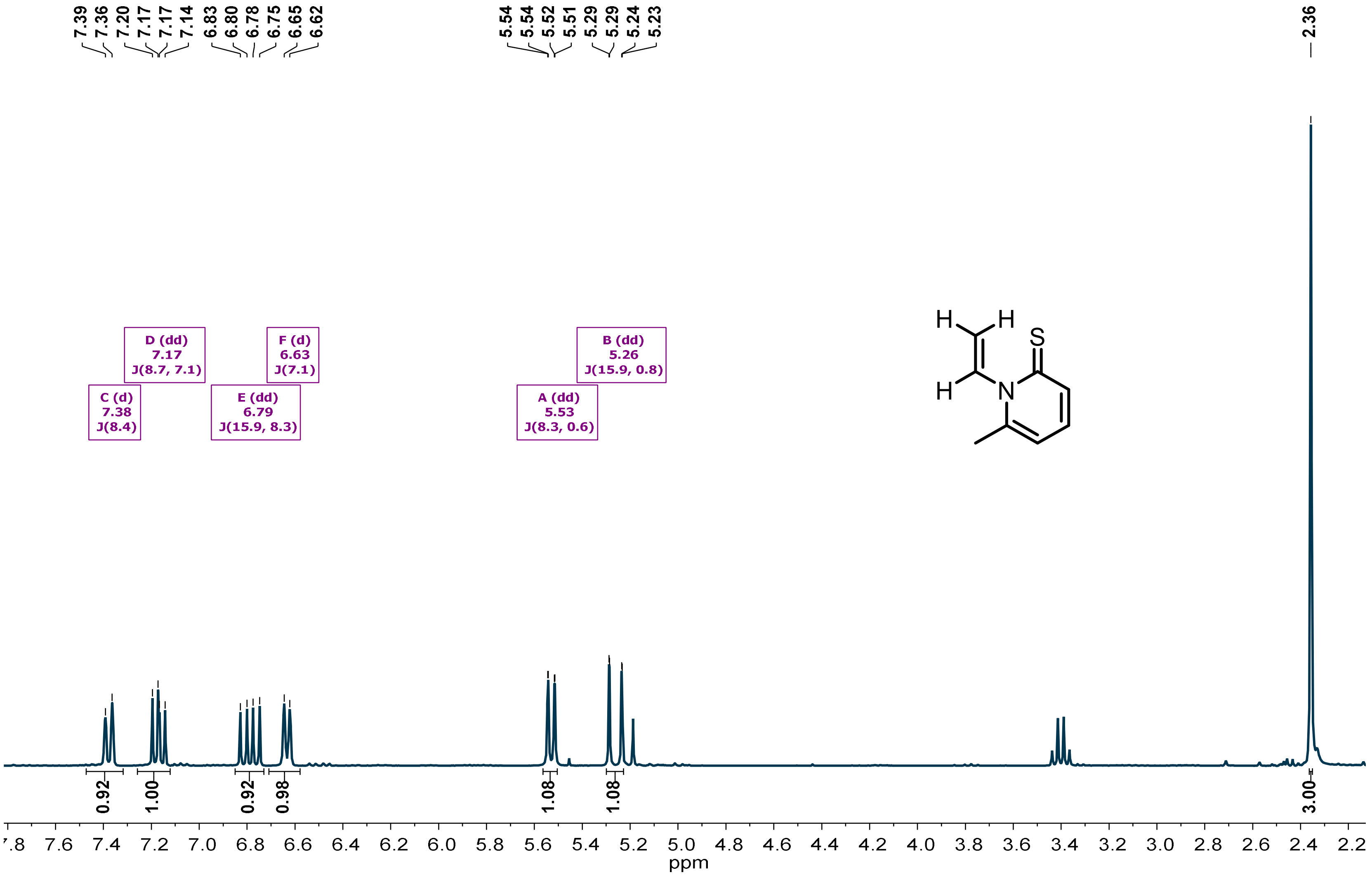

Figure S17. ^{1}H NMR spectrum of 6-methyl-1-vinylpyridine-2(1*H*)-thione in CD_3CN.

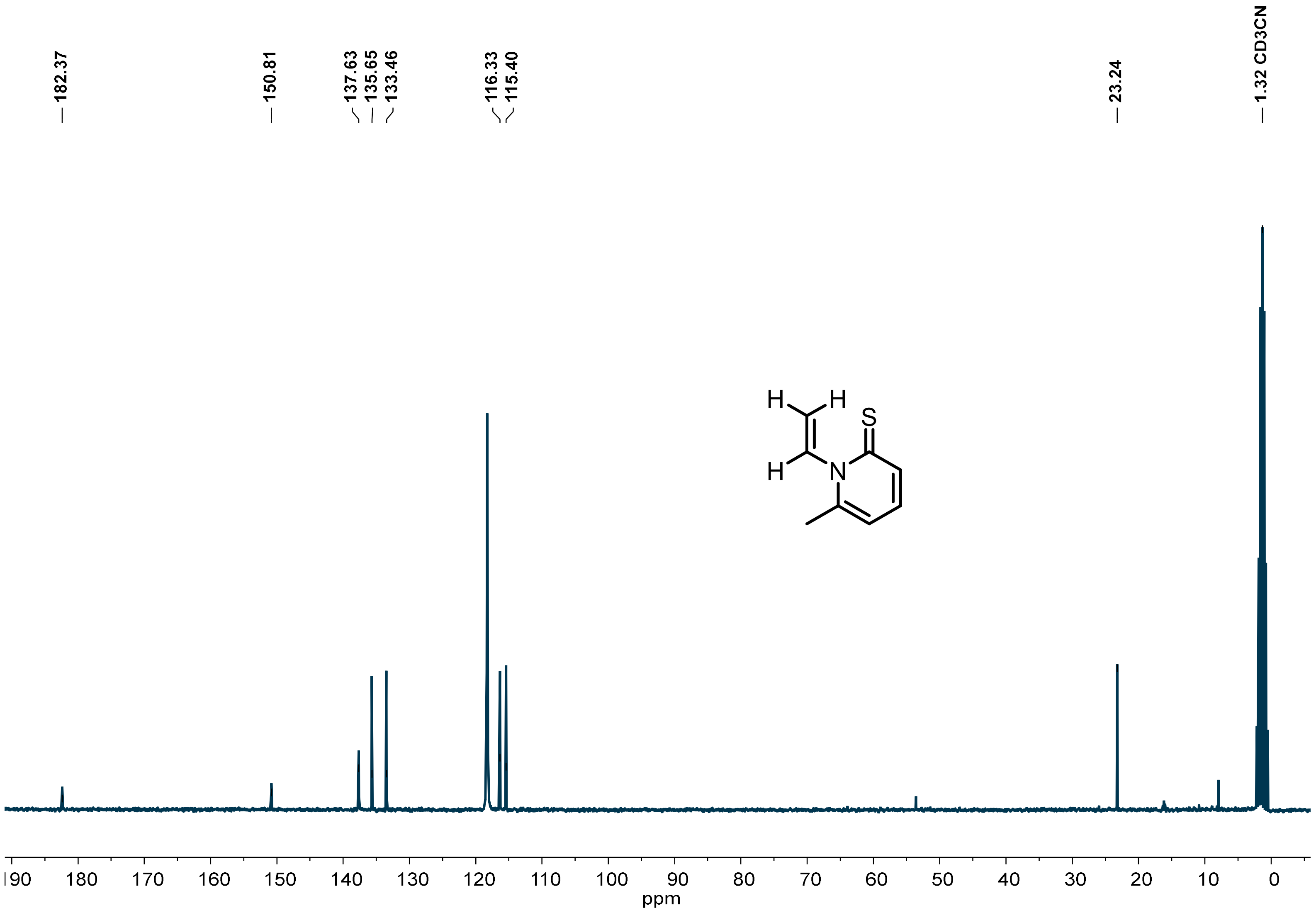

Figure S18. ^{13}C NMR spectrum of 6-methyl-1-vinylpyridine-2(1*H*)-thione in CD_3CN.

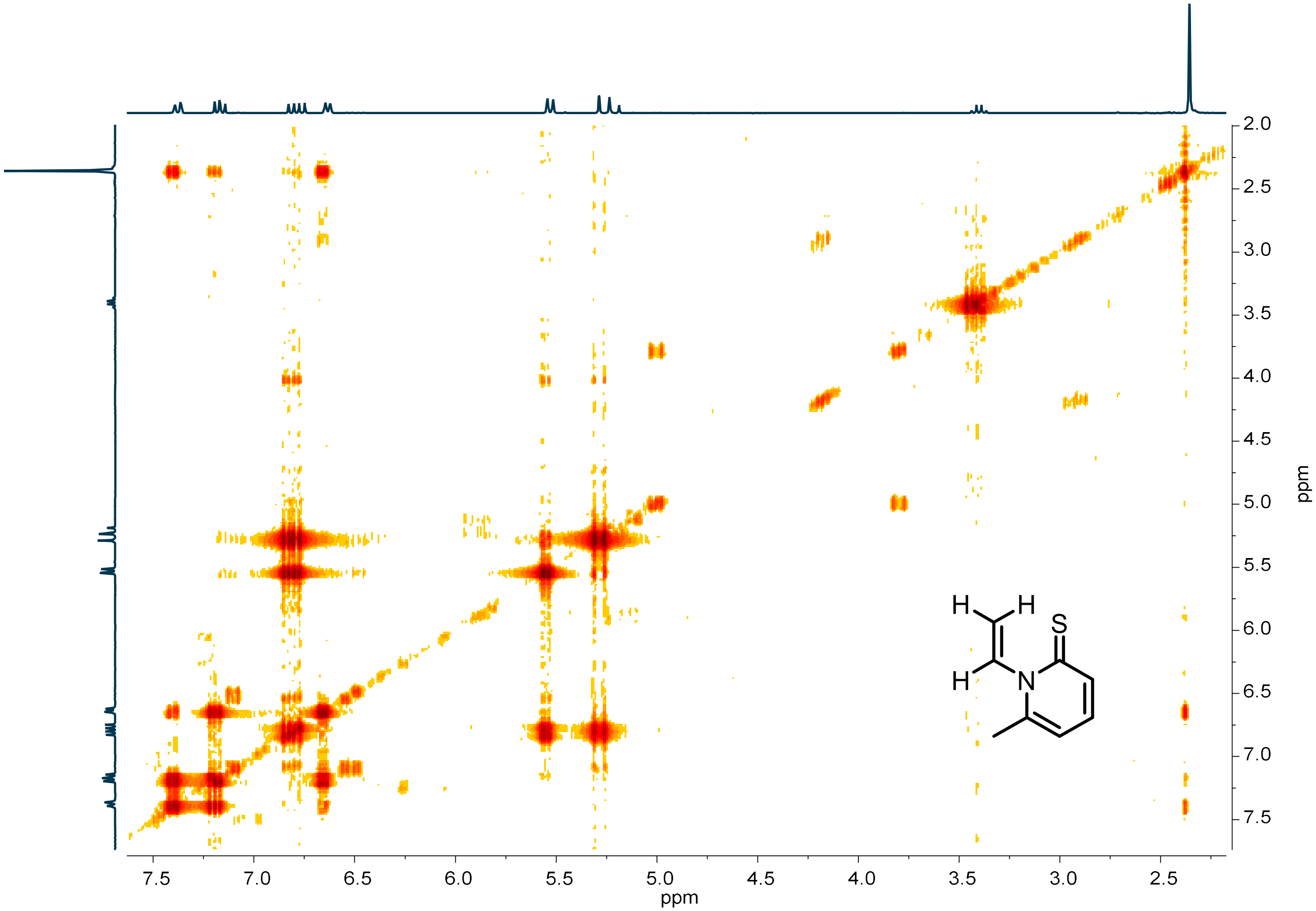

Figure S19. COSY spectrum of 6-methyl-1-vinylpyridine-2(1*H*)-thione in CD_3CN.

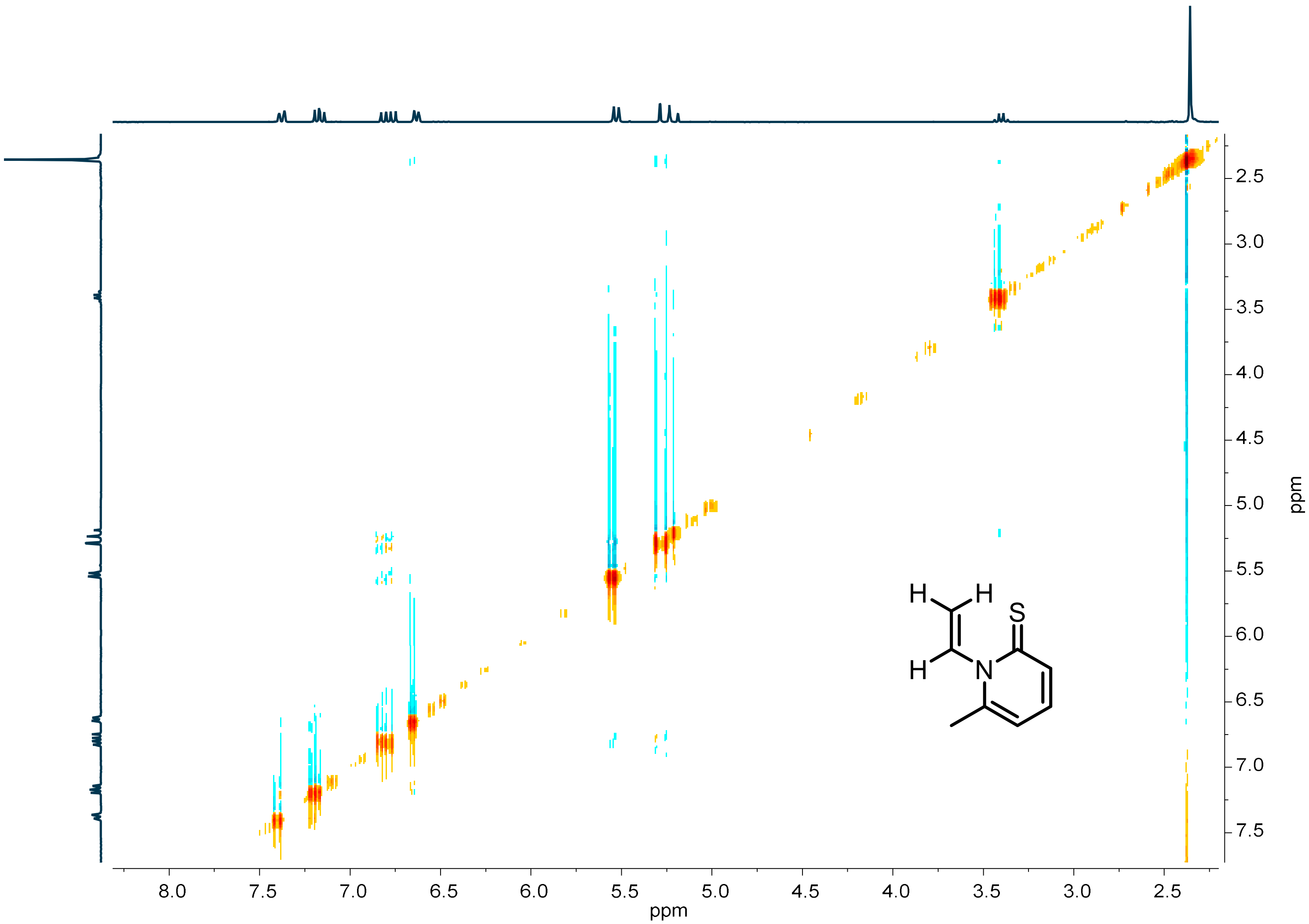

Figure S20. NOESY spectrum of 6-methyl-1-vinylpyridine-2(1*H*)-thione in CD_3CN.

3 Computational Details

The computational results presented have been achieved using the Vienna Scientific Cluster (VSC). Calculations were performed using the GAUSSIAN 09 software package[4] and the PBE0 functional without symmetry constraints. That functional uses a hybrid generalized gradient approximation (GGA), including 25% mixture of Hartree-Fock[5] exchange with DFT[6] exchange-correlation, given by Perdew, Burke and Ernzerhof functional (PBE).[7,8] The optimized geometries were obtained with the Stuttgart/Dresden ECP (SDD) basis set[9–11] to describe the electrons of the molybdenum atoms. For all other atoms, a standard 6-31G** basis set was employed.[12–17] A Natural Population Analysis (NPA)[18–24] and the resulting Wiberg indices[25] were used to study the electronic structure and bonding of the optimized species. Three-dimensional representations of the orbitals were obtained with Molekel.[26]

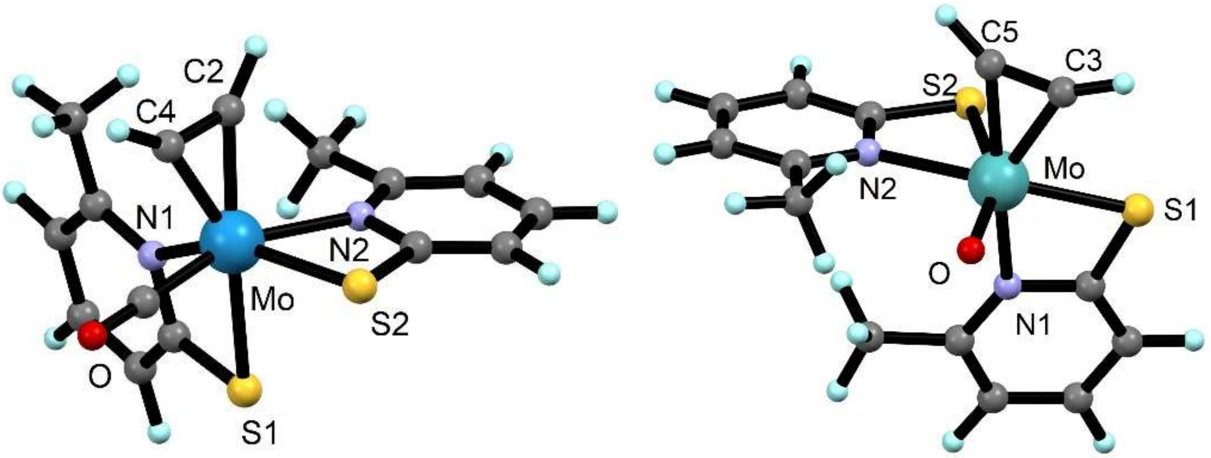

Figure S21. Optimized structures of **1** (left) and **2** (right).

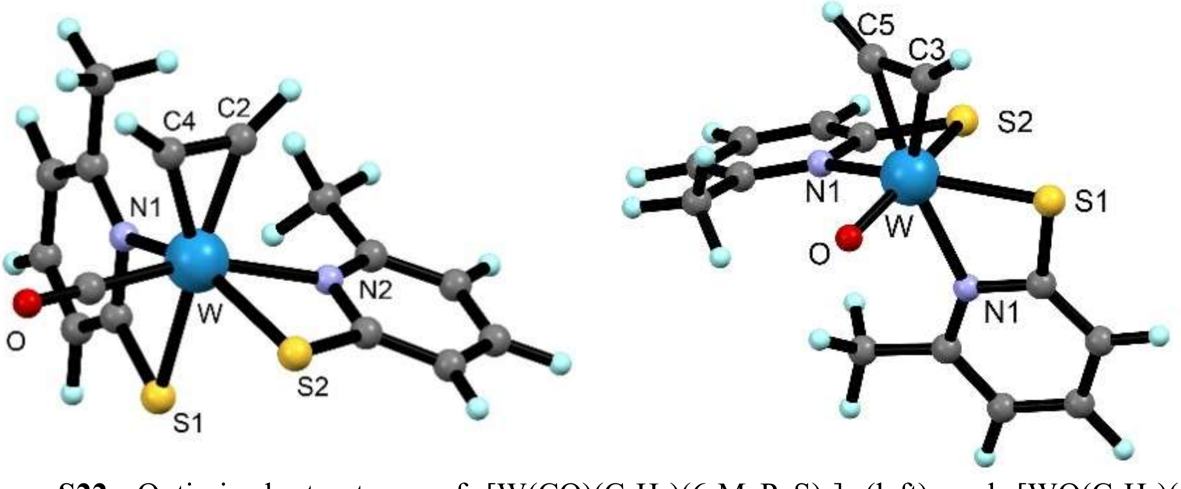

Figure S22. Optimized structures of $[W(CO)(C_2H_2)(6\text{-}MePyS)_2]$ (left) and $[WO(C_2H_2)(6\text{-}MePyS)_2]$ (right).

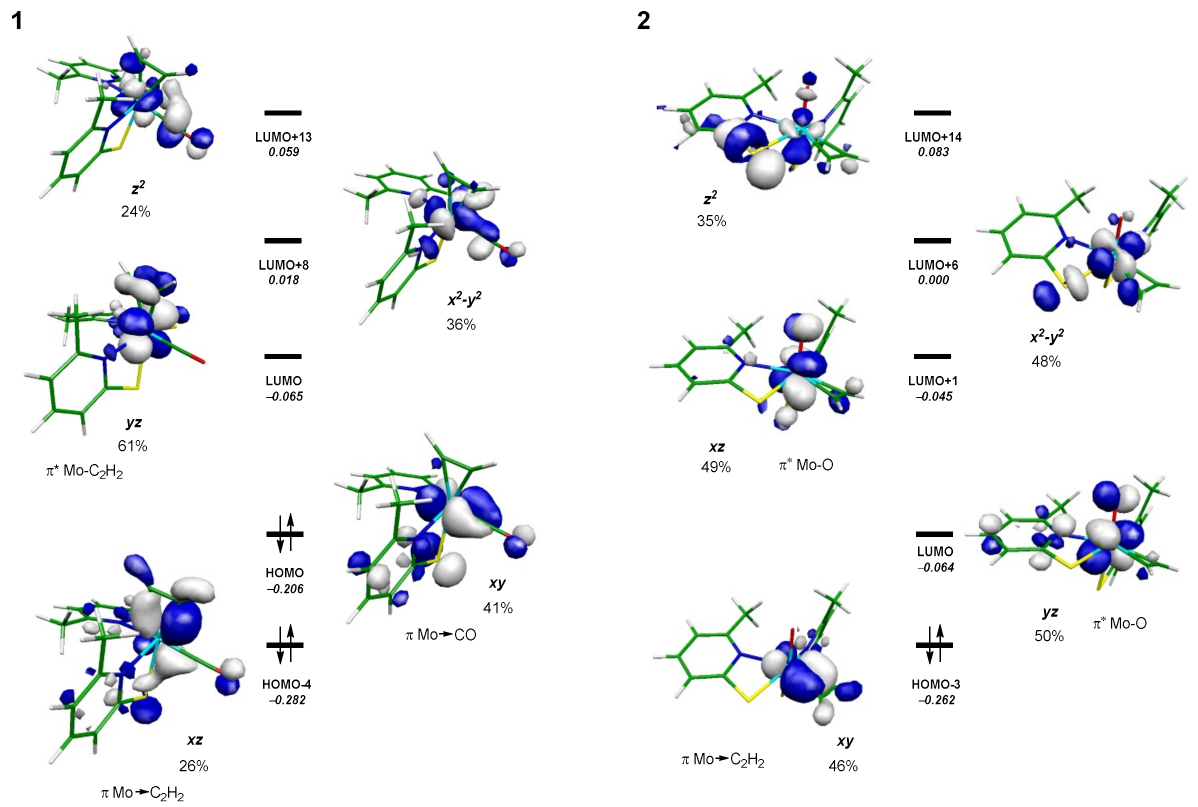

Figure S23. Frontier orbitals (d-splitting) for **1** (left) and **2** (right). Energy values are given in atomic units (italics).

Table S7. NPA charges of all atoms in **1**, **2**, [W(CO)(C_2H_2)(6-MePyS)$_2$], and [WO(C_2H_2)(6-MePyS)$_2$]. The atomic numbering scheme is the same as in Figures S21 and S22.

**[Mo(CO)(C_2H_2)(6-MePyS)$_2$] (1)**			**[MoO(C_2H_2)(6-MePyS)$_2$] (2)**			**[W(CO)(C_2H_2)(6-MePyS)$_2$]**			**[WO(C_2H_2)(6-MePyS)$_2$]**		
**Atom**	**Nr**	**NPA Charge**	**Atom**	**Nr**	**NPA Charge**	**Atom**	**Nr**	**NPA Charge**	**Atom**	**Nr**	**NPA Charge**
Mo	1	0.11351	Mo	1	0.87201	W	1	0.32767	W	1	1.12054
C	2	-0.3024	O	2	-0.49876	C	2	-0.3614	O	2	-0.60134
H	3	0.25484	C	3	-0.28462	H	3	0.25344	C	3	-0.33522
C	4	-0.27342	H	4	0.26587	C	4	-0.31399	H	4	0.26032
H	5	0.26383	C	5	-0.29245	H	5	0.26243	C	5	-0.34561
C	6	0.63749	H	6	0.26298	C	6	0.57833	H	6	0.25629
O	7	-0.4662	S	7	0.02727	O	7	-0.46881	S	7	0.01475
S	8	-0.04946	N	8	-0.50766	S	8	-0.05473	N	8	-0.52366
N	9	-0.49862	C	9	0.09036	N	9	-0.51727	C	9	0.09196
C	10	0.06624	C	10	-0.29483	C	10	0.06621	C	10	-0.29517
C	11	-0.29725	H	11	0.27037	C	11	-0.29773	H	11	0.27097
H	12	0.26974	C	12	-0.18033	H	12	0.27043	C	12	-0.17856
C	13	-0.19386	H	13	0.26034	C	13	-0.19481	H	13	0.26078
H	14	0.2583	C	14	-0.30124	H	14	0.25875	C	14	-0.30145
C	15	-0.31396	H	15	0.25883	C	15	-0.31187	H	15	0.2593
H	16	0.25641	C	16	0.27915	H	16	0.25716	C	16	0.28052
C	17	0.25064	C	17	-0.74804	C	17	0.25063	C	17	-0.74854
C	18	-0.74096	H	18	0.2712	C	18	-0.74247	H	18	0.27207
H	19	0.27035	H	19	0.25611	H	19	0.27206	H	19	0.25707
H	20	0.25704	H	20	0.27419	H	20	0.25764	H	20	0.27325
H	21	0.25975	S	21	-0.11679	H	21	0.26093	S	21	-0.12008
S	22	0.0733	N	22	-0.51629	S	22	0.05088	N	22	-0.53624
N	23	-0.4996	C	23	0.05356	N	23	-0.51501	C	23	0.05754
C	24	0.08437	C	24	-0.29991	C	24	0.08639	C	24	-0.30038
C	25	-0.2942	H	25	0.27016	C	25	-0.29508	H	25	0.27095
H	26	0.26995	C	26	-0.19031	H	26	0.27054	C	26	-0.18823
C	27	-0.18564	H	27	0.25865	C	27	-0.18389	H	27	0.25936
H	28	0.25925	C	28	-0.31727	H	28	0.2597	C	28	-0.31648
C	29	-0.30403	H	29	0.25809	C	29	-0.30443	H	29	0.25912
H	30	0.25721	C	30	0.26047	H	30	0.25782	C	30	0.26398
C	31	0.26945	C	31	-0.75029	C	31	0.27149	C	31	-0.75232
C	32	-0.74264	H	32	0.28178	C	32	-0.74375	H	32	0.28466
H	33	0.27622	H	33	0.25251	H	33	0.27591	H	33	0.25284
H	34	0.25483	H	34	0.27491	H	34	0.25582	H	34	0.27701
H	35	0.25954				H	35	0.26099			

4 References

(1) Sheldrick, G. M. A short history of SHELX. *Acta Cryst. A* **2008**, *64* (Pt 1), 112–122.

(2) Sheldrick, G. M. Crystal structure refinement with SHELXL. *Acta Cryst. C* **2015**, *71* (Pt 1), 3–8.

(3) Johnson, C. K. *ORTEP. Report ORNL-3794.*, 1965.

(4) Gaussian 09, Revision A.01, M. J. Frisch, G. W. Trucks, H. B. Schlegel, G. E. Scuseria, M. A. Robb, J. R. Cheeseman, G. Scalmani, V. Barone, B. Mennucci, G. A. Petersson, H. Nakatsuji, M. Caricato, X. Li, H. P. Hratchian, A. F. Izmaylov, J. Bloino, G. Zheng, J. L. Sonnenberg, M. Hada, M. Ehara, K. Toyota, R. Fukuda, J. Hasegawa, M. Ishida, T. Nakajima, Y. Honda, O. Kitao, H. Nakai, T. Vreven, J. A. Montgomery, Jr., J. E. Peralta, F. Ogliaro, M. Bearpark, J. J. Heyd, E. Brothers, K. N. Kudin, V. N. Staroverov, R. Kobayashi, J. Normand, K. Raghavachari, A. Rendell, J. C. Burant, S. S. Iyengar, J. Tomasi, M. Cossi, N. Rega, J. M. Millam, M. Klene, J. E. Knox, J. B. Cross, V. Bakken, C. Adamo, J. Jaramillo, R. Gomperts, R. E. Stratmann, O. Yazyev, A. J. Austin, R. Cammi, C. Pomelli, J. W. Ochterski, R. L. Martin, K. Morokuma, V. G. Zakrzewski, G. A. Voth, P. Salvador, J. J. Dannenberg, S. Dapprich, A. D. Daniels, Ö. Farkas, J. B. Foresman, J. V. Ortiz, J. Cioslowski, D. J. Fox, Gaussian, Inc., Wallingford CT, **2009**.

(5) Hehre, W. J.; Radom, L.; Schleyer, P. v. R.; Pople, J. A. *Ab Initio Molecular Orbital Theory*; Wiley, 1986.

(6) Parr, R. G.; Yang, W. *Density Functional Theory of Atoms and Molecules*; Oxford University Press, 1989.

(7) Perdew, J. P. Density-functional approximation for the correlation energy of the inhomogeneous electron gas. *Phys. Rev. B* **1986**, *33* (12), 8822–8824.

(8) Perdew, J. P.; Burke, K.; Ernzerhof, M. Generalized Gradient Approximation Made Simple [Phys. Rev. Lett. 77, 3865 (1996)]. *Phys. Rev. Lett.* **1997**, *78* (7), 1396.

(9) Leininger, T.; Nicklass, A.; Stoll, H.; Dolg, M.; Schwerdtfeger, P. The accuracy of the pseudopotential approximation. II. A comparison of various core sizes for indium pseudopotentials in calculations for spectroscopic constants of InH, InF, and InCl. *J. Chem. Phys.* **1996**, *105* (3), 1052–1059.

(10) Küchle, W.; Dolg, M.; Stoll, H.; Preuss, H. Energy-adjusted pseudopotentials for the actinides. Parameter sets and test calculations for thorium and thorium monoxide. *J. Chem. Phys.* **1994**, *100* (10), 7535–7542.

(11) Haeussermann, U.; Dolg, M.; Stoll, H.; Preuss, H. Accuracy of energy-adjusted quasi-relativistic ab initio pseudopotentials – all-electron and pseudopotential benchmark calculations for HG, HGH and their cations. *Mol. Phys.* **1993**, *78*, 1211–1224.

(12) Curtiss, L. A.; McGrath, M. P.; Blaudeau, J.-P.; Davis, N. E.; Binning, R. C.; Radom, L. Extension of Gaussian-2 theory to molecules containing third-row atoms Ga–Kr. *J. Chem. Phys.* **1995**, *103* (14), 6104–6113.

(13) McGrath, M. P.; Radom, L. Extension of Gaussian-1 (G1) theory to bromine-containing molecules. *J. Chem. Phys.* **1991**, *94* (1), 511–516.

(14) Raghavachari, K.; Trucks, G. W. Highly correlated systems. Excitation energies of first row transition metals Sc–Cu. *J. Chem. Phys.* **1989**, *91* (2), 1062–1065.

(15) Hay, P. J. Gaussian basis sets for molecular calculations. The representation of 3 d orbitals in transition-metal atoms. *J. Chem. Phys.* **1977**, *66* (10), 4377–4384.

(16) Krishnan, R.; Binkley, J. S.; Seeger, R.; Pople, J. A. Self-consistent molecular orbital methods. XX. A basis set for correlated wave functions. *J. Chem. Phys.* **1980**, *72* (1), 650–654.

(17) McLean, A. D.; Chandler, G. S. Contracted Gaussian basis sets for molecular calculations. I. Second row atoms, Z =11–18. *J. Chem. Phys.* **1980**, *72* (10), 5639–5648.

(18) Reed, A. E.; Curtiss, L. A.; Weinhold, F. Intermolecular Interactions from a Natural Bond Orbital, Donor-Acceptor Viewpoint. *Chem. Rev.* **1988**, *88*, 899–926.

(19) Reed, A. E.; Weinstock, R. B.; Weinhold, F. Natural population analysis. *J. Chem. Phys.* **1985**, *83* (2), 735–746.

(20) Reed, A. E.; Weinhold, F. Natural localized molecular orbitals. *J. Chem. Phys.* **1985**, *83* (4), 1736–1740.

(21) Reed, A. E.; Weinhold, F. Natural bond orbital analysis of near-Hartree–Fock water dimer. *J. Chem. Phys.* **1983**, *78* (6), 4066–4073.

(22) Foster, J. P.; Weinhold, F. Natural Hybrid Orbitals. *J. Am. Chem. Soc.* **1980**, *102*, 7211–7218.

(23) Carpenter, J. E. Extension of Lewis structure concepts to open-shell and excited-state molecular species. PhD thesis, University of Wisconsin, Madison, WI, 1987.

(24) Carpenter, J. E.; Weinhold, F. Analysis of the geometry of the hydroxymethal radical by the "Different hybrids for different soins" natural bond orbital procedure. *J. Mol. Struct. (Theochem)* **1988**, *169*, 41–62.

(25) Wiberg, K. B. Application of the Pople-Santry-Segal CNDO Method to the Cyclopropylcarbinyl and Cyclobutyl Cation and to Bicyclobutane. *Tetrahedron* **1968**, *24*, 1083–1096.

(26) Portmann, S.; Lüthi, H. P. MOLEKEL: An Interactive Molecular Graphics Tool. *CHIMIA* **2000**, *54* (12), 766–770.
